# Environmental impact assessment of transportation and land alteration using Earth observational datasets: Comparative study between cities in Asia and Europe

**DOI:** 10.1016/j.heliyon.2023.e19413

**Published:** 2023-08-28

**Authors:** Khalid Hardan Mhana, Shuhairy Bin Norhisham, Herda Yati Binti Katman, Zaher Mundher Yaseen

**Affiliations:** a Institute of Energy Infrastructure (IEI) and Department of Civil Engineering, College of Engineering, Universiti Tenaga Nasional (UNITEN), Putrajaya Campus, Jalan IKRAM-UNITEN, 43000 Kajang, Selangor, Malaysia; b Department of Civil Engineering, College of Engineering, Universiti Tenaga Nasional (UNITEN), Putrajaya Campus, Jalan IKRAM-UNITEN, 43000 Kajang, Selangor, Malaysia; c Civil Engineering Department, College of Engineering, University Of Anbar, Iraq; d Civil and Environmental Engineering Department, King Fahd University of Petroleum & Minerals, Dhahran, 31261, Saudi Arabia; e Interdisciplinary Research Center for Membranes and Water Security, King Fahd University of Petroleum & Minerals, Dhahran 31261, Saudi Arabia

**Keywords:** Geospatial approaches, Kernel density estimation, Transportation accessibility, Heat island impact, Surface energy budget, Landsat science

## Abstract

Developments in the transportation field are emerging because of the growing worldwide demand and upgrading requirements. This study measured the transportation development, shortage distance, and decadal land transformation of Kuala Lumpur and Madrid using various remote sensing and GIS approaches. The kernel density estimation (KDE) tool was applied for road and railway density analysis, and hotspot information increased the knowledge about assessable areas. Landsat datasets were used (1991–2021) for land transformation and related analyses. The built-up land increased by 1327.27 and 404.09 km^2^ in Kuala Lumpur and Madrid, respectively. In the last thirty years, the temperature increased 6.45 °C in Kuala Lumpur and 4.15 °C in Madrid owing to urban expansion and road construction. Chamberi, Retiro, Moratalaz, Salama, Wangsa Maju, Titiwangsa, Bukit Bintang, and Seputeh have very high road densities. KDE measurements showed that the road densities in Kuala Lumpur (4498.34) and Madrid (9099.15) were high in the central parts of the city, and the railway densities were 348.872 and 2197.87, respectively. The observed P values were 0.99 and 0.96 for traffic signals and 0.98 and 0.99 for bus stops, respectively. The information provided by this study can support local planners, administrators, scientists, and researchers in understanding the global transportation issues that require implementation strategies for ensuring sustainable livelihoods.

## 1 Introduction

### 1.1 Research background

Network analysis is essential for addressing traffic accidents, jams, shortage route management, and related issues because of the socioeconomic significance of road accidents and other effects created by the development of transportation systems worldwide [1,2]. Globally, 518 billion USD are spent owing to road traffic accidents (RTA); therefore, the management of the transportation sector is essential. The Global Status Report of the World Health Organization (WHO) presents the safety report of roads based on the annual number of casualties per 100,000 people and, for example, the rate of casualties due to road traffic collisions (RTCs) in the Kingdom of Saudi Arabia has increased from 17.4 to 27.4 in the last years (https://www.who.int/news-room/fact-sheets/detail/road-traffic-injuries). This cumulative accident frequency is the highest among the countries in the region, and it is clearly higher than the accident rates in other G-20 nations [3]. Transport infrastructure networks (TINs) and land use and land cover (LULC) are closely interwoven, which is supported hypothetically by the land use and transport response sequence [4,5]. The development of a TIN can promote regional efficiency and generate new urban development opportunities. Furthermore, global urbanization may result in the development of regional transport associations and expansion of TINs [[6], [7], [8]]. Certain connections are straight and comparatively fast, while others require extended periods of time. Although the interrelationship between LULC and TINs has been the subject of several reviews in recent decades, studies on other trends have been considerably incomplete, particularly those that are extended in nature [6]. Insufficient literature is available on the sequence of land occupation, and thus, the long-term influence of TINs on LULC was the focus of this study. In the analysis of the current empirical literature, this study examined whether the magnitude of LULC transformation can be explained by TIN enlargement. It is clear that LULC and TIN are subjected to other external influences [[9], [10], [11]].

According to the Malaysia Department of Statistics report (http://www.citypopulation.de/), the population of the Greater Kuala Lumpur grew from approximately 2.4 million inhabitants in 1991 to 5.5 million in 2010. This number represented approximately 20% of the predicted Malaysian population for the year 2010, 28.25 million inhabitants. The detailed evidence related to the nature and scope of land occupation and their variations during a certain period is accumulative, especially at the municipal level [12,13]. A review of the spatial evolution of the Earth is mainly aimed at understanding the effect of the anthropogenic activities on the natural environment along dissimilar time periods. The evidence provided by remote sensing (RS) satellites on the Earth's topography and substructures shows alterations in their development [14]. Image-to-image comparisons and map-to-map assessments reveal the influence of the LULC transformation process. Research on LULC transformation depends to a large extent on the development of the scientific RS field [15].

### 1.2 Relevant research

The road network system in Malaysia faces numerous difficulties, according to the green logistics agencies. The main difficulties acknowledged are the convenience and connectivity of the road network system in Port Klang, which is “last mile connectivity” for cargo [16]. Rapid infrastructure development has resulted in land alteration, heat island effects, and transportation-related issues worldwide. Strategies to control these phenomena are essential for a sustainable development. In addition to LULC variations, urban expansion has become a primary topic of discussion in anthropology [[17], [18], [19]]. A road network can cause a reduction in areas with vegetation, climate variation, groundwater shortages, and air pollution; therefore, examination of the environmental impact and other road construction-related issues is essential to prevent unexpected developments, build awareness, and reduce deforested areas. Economic growth and population pressure are among the factors that produce rapid urban expansion. Lack of preparation for LULC transformation management at the municipal level affects the local environment and zonal climate, which can lead to expansion of the urban heat island (UHI). The association between transportation networks and urban growth is at the center of discussions about development, sustainability, cohesion, regionalization, and core–periphery relation. Furthermore, the search for accessibility has been a driving factor of multifaceted projects and the appearance of subcenters [20]. In 1981, the position distribution with advanced convenience standards were gathered in the principal cummerbund everywhere in Madrid, which might be understood as an incomplete number of residences captivating fragments in the metropolitan procedures [21]. The transportation corridors were the focus of attention during this period, and an individual south-western subcenter cluster appeared. In 2011, the situation was comparable, but the number of subcenters increased. Nonetheless, the northern areas did not advance as much as the southern ones, undoubtedly because new infrastructure just started to be built and was less developed than in the southern direction. In 2011, the land occupied in the southern belt was notable. Thus, additional experimental methods have been applied to mobility in order to disentangle polycentric constructions. Consequently, research on LULC transformation at specific locations might help to comprehend the spatial magnitude and degree of the variations, in addition to other anthropology-related ecological fluctuations [22,23]. Although Malaysia has extensively used satellite images in research, additional analysis is still required to understand the multi-temporal LULC in the Kuala Lumpur metropolitan city.

Land surface temperature (LST) is an important indicator of the Earth's surface energy budget when measuring LULC fluctuations and additional transformations on the Earth's surface [17,23,24]. Using RS and GIS approaches to investigate and examine extreme variations, numerous studies have established that LULC affects LST [15,[25], [26], [27], [28]]. Kuala Lumpur city experiences an equatorial climatic change throughout the year. It has a yearly thermal fluctuation between 17 and 38 °C and a regular everyday mean LST of approximately 28 °C, with minimum and maximum temperatures of 23 and 32 °C, respectively [29,30]. The aforementioned research investigated the Greater Kuala Lumpur from 1996 to 2013. In this period, there has been an extreme transformation of farmlands, forests, water bodies, and built-up areas. Water bodies and forests were reduced by approximately 48.72% and 37.59%, respectively [29]. Researchers have discovered that in the past decades, the formation of new transportation substructures has been an important factor in the provincial development of Madrid. The highway and road network densities increased by 187%, and 218%, respectively [31]. In a green scenario, the industrial and commercial urban areas will increase by 18% and 16%, respectively. Arable land will contract by 16%, whereas mixed agriculture will increase somewhat (0.50%). Urban expansion has taken place in the south-east of Madrid city, accompanied by highways and circular roads [32]. Other geospatial indicators, including the normalized difference vegetation index (NDVI), normalized difference moisture index (NDMI), soil-adjacent vegetation index (SAVI), normalized difference built-up index (NDBI), urban heat island (UHI), and urban thermal field variation index (UTFVI) are essential for the analysis of environmental variation.

### 1.3 Research motivation and aim

The development of transportation and expansion of the road network in the urban–fringe–rural position is intensifying, and relevant studies are indispensable for developers and policymakers [4,33]. Analyses on the interrelation between road infrastructure and ecological problems are limited; therefore, further studies can help researchers, scientists, and administration teams improve the application of new approaches on a specific domain. The aforementioned studies indicate that road-related accidents are an additional risk [34]. GIS-based approaches can help future disaster-related decision-making with improvements in spatial analyses using short-term epochs [35,36]. There are particular restrictions on the road network analysis system, LULC-related evidence, and ecological influences on the Earth's surface. Traffic volume estimation [[37], [38], [39]], road density analysis [5,40,41], shortage distance, accident-prone area analysis [42], suitable sites, and numerous LULC singularities are associated with land use, which is why road network analysis or review-related presentations are interwoven with LULC. The objective of this research is to (a) determine the LULC alteration of Kuala Lumpur (Malaysia) and Madrid (Spain) based on analyses of changes in vegetation and built-up areas; (b) use geospatial indices such as NDVI, SAVI, NDBI, and NDMI for understanding the land alteration and its impact on the environment; (c) utilize UHI and UTFVI for measuring thermal variations and ecological fluctuation in those major cities; and (d) use road network-related GIS applications such as kernel density estimation (for both roads and railways). This information is essential for city planning to ensure a sustainable and healthy life. Because current transportation development and the worldwide heat island effect are harmful to livelihoods, proper planning, management, and novel techniques can help reduce these issues. Recent road-related observations and transportation information might help to reduce traffic congestion and air pollution caused by vehicles, promote healthy vegetation, and achieve an overall reduction in the environmental impact over the selected study areas [43]. This investigation also helps with decision-making regarding heat island effects, ecological alteration, and land transformation, along with transportation construction-related issues, in the major cities of the two selected countries.

## 2 Description of study areas

**Fig. 1:**
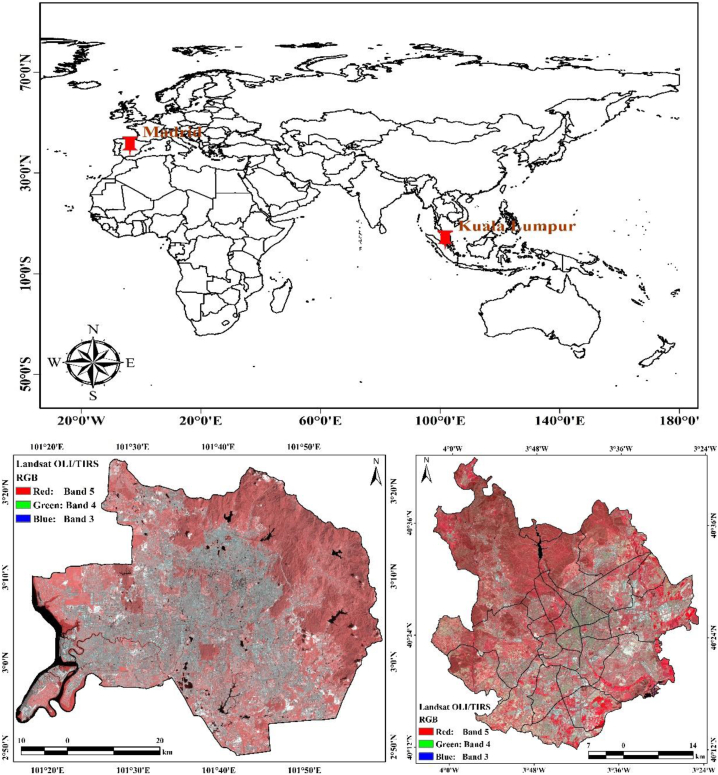
Locational map of this selected study area.

The cities investigated were Kuala Lumpur and Madrid, which are situated in Malaysia and Spain, respectively. Madrid city has an extension of approximately 1759 km^2^, a population of approximately 6,751,000, and is the most compactly inhabited province in Spain. The investigated city of Madrid extends from 4° 08′ W to 3° 22′ W and from 40° 12′ N to 40° 50′ N. It generates approximately 17% of the country's gross domestic product (GDP) and is the second most significant manufacturing hub in Spain. It is the main node for airport travel between European and Latin American cities. Its conserved green spaces and densely forested land, which comprise approximately 12% and 9% of the total area, respectively, defend the water resources and soil. Approximately 48% of the forest areas are protected, whereas the natural threatened expanses comprise approximately 40.51% of the provincial zone (https://land.copernicus.eu/pan-european/corine-land-cover/clc-2006). Since 2008, Madrid city has experienced a rapid socioeconomic development, which has led to outsized alterations and widespread LULC variations, numerous of which are connected to the road network system. This development has exerted a notorious influence throughout the country [44]. In 2010, the Spain Observatory of Sustainability affirmed that the period between 2000 and 2006 might have seen the widest infrastructure development and urbanization in Spain's history, and the city of Madrid was one of the cities where these developments had the greatest impacts. The Madrid region should be measured for its multidimensional urban development characteristics. Because of its multifaceted expansion, the city is one of the largest urban zones in the Mediterranean area [45], with a specific process of urban sprawl. Madrid city's spatial development is guided by municipal and provincial planning; regional strategies are applicable across the different zones and consider the complete area, whereas municipal strategies focus on the municipalities, cities, and towns and are compulsory in these areas. The metropolitan area is divided into 179 districts (Fig. 1). A comprehensive investigation of the arrangement of each municipality falls outside the scope of this analysis because it addresses a larger scale. The average annual temperature varies between 6 °C in the highlands and 15 °C in the basins. Semi natural zones and forest lands comprise approximately 48% of the entire landmass, inside which shrub and/or herbaceous vegetation associations characterize approximately 78% and forests characterize 20% of the landmass. Deciduous forests are present in approximately 40% of the vegetated land.

For Kuala Lumpur city, the investigated area comprises approximately 3030 km^2^ and is bounded by the Titiwangsa Mountains in the north and east, Port Dickson in the south, and the Straits of Malacca in the west part. Kuala Lumpur extends from 101° 15′ E to 101° 58′ E and from 2° 45′ N to 3° 24′ N. The Kuala Lumpur metropolitan city is Malaysia's commercial and industrial hub, as described in the 2014 plan “Developing Greater Kuala Lumpur/Klang Valley as an Engine of Economic Growth” (https://esd.ny.gov/2014-economic-transformation-program). With approximately 7.5 million inhabitants, it has attracted foreign laborers, mainly from Nepal, Indonesia, Bangladesh, India, and Myanmar, and is the fastest developing municipal province in Malaysia from the economic and population viewpoints. Kuala Lumpur is expanding to the north towards Rawang and to the south in the direction of the Negeri Sembilan boundary [46]. Kuala Lumpur city experiences an equatorial extreme climatic condition through the year, with the annual temperature fluctuating between 17 and 38 °C and a regular everyday mean temperature of approximately 28 °C with a maximum and minimum temperatures of 32 and 23 °C, respectively. The regular annual relative humidity varies between 70% and 90%, with regular-year rainfall recorded at approximately 223 cm/year [29]. Throughout the monsoonal wind season, the inhabitants experience sunshine throughout the day but precipitation at the sunset. The rainy periods commonly occur from April to June and from October to December.

## 3 Materials and methods

### 3.1 Applied datasets and information

**Fig. 2:**
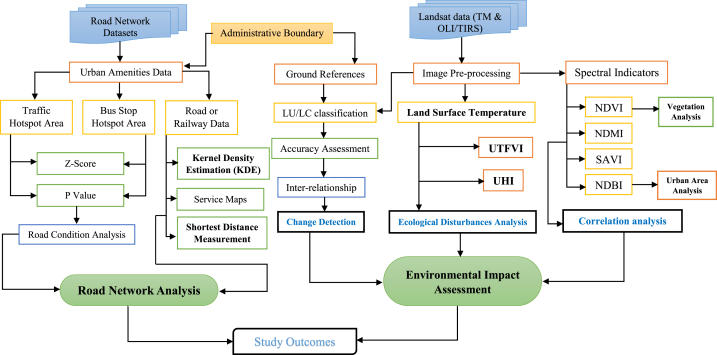
Methodological flowchart of this investigation.

The examination of LULC alterations necessitates a considerable quantity of Earth's observational datasets to conduct an operative investigation. Earth observational datasets function as countless data sources from which rationalized LULC maps and variations can be examined based on simulations. Different datasets have been applied to investigate the environmental issues and ecological alteration in two major world cities, Kuala Lumpur, Malaysia, and Madrid, Spain, from 1991 to 2021. Optical datasets are widely applied for landmass change analysis, in addition to environmental issues such as vegetation damage, urban expansion, moisture losses, heat variation, and temperature fluctuations. Two different datasets were used in this investigation: Landsat 5 TM and Landsat 8 OLI/TIRS, which were derived from the USGS website (https://earthexplorer.usgs.gov/) and an open-source platform for identifying network-related information worldwide. These datasets were used for road and railway density estimation, shortage distance measurement, and hotspot analysis of bus and traffic signals. Furthermore, satellite images of four different dates were used to calculate the environmental fluctuations from 1991 to 2021 with ten years of interval. Table 1 lists the overall data acquisition information for the satellite-use dates. ArcGIS software version 10.8 and ERDAS Imagine (version 2014) were used for the classification, data analysis, density estimation, and map layout (Fig. 2).

**Table 1:** Details about the data information and acquisition date.

Kuala Lumpur				
Satellite Sensor	Acquiring Date	Path/Row	Cloud Cover	Website
Landsat 5 TM	21-02-1991	147; 043	<5%	https://earthexplorer.usgs.gov/
	12-09-2001	147; 043	<5%	
	06-07-2011	147; 043	<5%	
Landsat 8 OLI/TIRS	07-02-2021	147; 043	<5%	
**Madrid**				
Landsat 5 TM	26-05-1991	147; 043	<5%	https://earthexplorer.usgs.gov/
	29-05-2001	147; 043	<5%	
	07-04-2011	147; 043	<5%	
Landsat 8 OLI/TIRS	18-04-2021	147; 043	<5%

### 3.2 Satellite data pre-processing and classification

Satellite dataset preprocessing methods were applied to take advantage of the excellence and discernibility of the Earth's observational datasets. These methods provided the preliminary platform where satellite dataset geometric, atmospheric, radiometric, enhancement, and topographic corrections were completed to improve the satellite image quality [13,47]. ENVI version 5.6 software was used for fast line-of-sight atmospheric analysis of hypercubes (FLAASH) [48,49]. Subsequently, corrections to Earth observational satellite datasets were categorized according to various LULC features by applying a signature-based, supervised dataset classification procedure. Some techniques were applied for satellite dataset pre-processing contingent upon the imagery situation, discernibility, and excellence in each case [50]. LULC image classification methods label pixels based on preprocessed satellite datasets for the procurement of categorized images. Every pixel is measured as an exclusive object and allocated to a specific case of complete pixel-based image classification [51,52]. Object-based image classification is the information collected from a related pixel group, which classifies the pixels into graphical sizes, shapes, and additional spatial characteristics. Customarily disseminated datasets were anticipated underneath the parametric classifiers. The Landsat images are medium-resolution satellite datasets, with a pixel resolution of 30 m. Consequently, the classification methods were applied to the available evidence [52,53]. The used area formula was $\left(\frac{Count\times 900}{1000000}\right)$, where 900 was applied because of the 30 m spatial resolution of the Landsat TM and OLI/TIRS datasets.

Nonparametric image classifiers do not consider normal or statistical classification parameters. Spectral mixture analysis and the fuzzy methodology were adopted as operative approaches for sub-pixel-based satellite imagery classification because they assist in the categorization of LULC in a specific section [54]. Fuzzy approaches and supervised and unsupervised methods are typically applied to classify remotely sensed satellite images. Similarly, semi-supervised methods have been applied to LULC image classification. In this study, a supervised LULC classification algorithm was designed. Other classification methods, such as maximum likelihood classification, Mahalanobis distance, K-means clustering, regression splines method, iterative self-organizing data analysis, graph-based semi-supervised model, k-nearest neighbor, random forest classifier, parallelepiped, classification and regression tree (10.13039/100012513CART), fuzzy C-means (FCM) clustering, support vector machine classifiers, artificial neural networks, semi-supervised transductive support vector machine (TSVM), multivariate adaptive, minimum distance, spectral angular mapper classification, self-trained models, and semi@@ classification approaches, can be used not only for image classification but also for forecasting [55].

### 3.3 Post-processing applications

Post-classification of satellite imagery is an enhancement procedure, while improving the excellence of the LULC image-classified diagram allows noise removal and prevents misclassification mistakes [56]. This procedure assists in the achievement of complete accuracy by eliminating the scattered and single pixels contained in a particular LULC classification image. Post-classification dispensation contributes to resolving the misclassification mistake that occurs because of considerable spectral misunderstanding among LULC features (e.g., a misunderstanding between barren land and agricultural land). This procedure improves the reclassification of misclassified pixels [57]. LULC classification imagery was used to authenticate the modification investigation for dissimilar time intervals above a specific area. The LULC alteration examination was performed to identify variations that occurred in specific and precise parts, and for creating valuable statements related to environmental protection. The proportion and alteration rate were predicted to categorize the magnitude of the variations that occurred among dissimilar time intervals. Consequently, this study identified five of the foremost LULC categories. These include water bodies, dense vegetation, grass, open forests, built-up land, and agricultural land. Particular and inaccessible pixels are the least understandable and comprehensive in the furthermost communal adjacent feature. Consequently, this investigation functioned as a confusion matrix method for evaluating the classification accuracy [58,59]. Therefore, the user, producer, overall accuracy, and kappa coefficients were used for pattern satellite image classification and signature assortment trustworthiness. Table 2 lists the kappa coefficient scale and the strength of the kappa coefficient for satellite image classification. The accuracy assessment was realized using a method in which the pixel-based modification investigation was an experimental side-to-side modification recognition technique, where real variations and changeability were also distinguished using ArcGIS software (Table 2). Subsequently, accuracy assessment and area calculation were completed using ArcGIS software v10.8, with count values of the satellite-classified features (Eqs. (1), (2))).

(1) $$OA=\left(\frac{\sum_{i=1}^{k}n_{ij}}{n}\right)$$

(2) $$K_{i}=\frac{\left(Observed\;accuracy-Change\;accuracy\right)}{\left(1-Change\;accuracy\right)}$$

where $n_{ij}$ indicates the diagonal basis of the error matrix, k indicates the total number of LULC classes, and n is the total sample collection-based error matrix.

**Table 2:** Scale of Kappa coefficient.

Sl. No.	Value of K	Strength of agreement
1	<0.20	Poor
2	0.21–0.40	Fair
3	0.41–0.60	Moderate
4	0.61–0.80	Good
5	0.81–1.00	Very good

### 3.4 Land surface temperature measurement

#### 3.4.1 Landsat 5 TM

The LST of four decadal Earth observational datasets was applied to calculate the thermal variation of two different world cities, Kuala Lumpur and Madrid. The LST was determined using Landsat TM (band 6) and Landsat OLI/TIRS (band 10) [60]. A subsequent procedure was used to develop the LST maps of the study area [61]. The digital number (DN) of the spectral radiance [62] was estimated using the formula in Eq. (3).

(3) $$L=\left(\frac{L_{\max}-L_{\min}}{{DN}_{\max}}\right)\times Band+L_{\max}$$

where L is the spectral radiance (SR), $L_{\min}$ is 1.238 (the spectral radiance when the DN value is 1), $L_{\max}$ is 15.6000 (the spectral radiance when the DN value is 255), and ${DN}_{\max}$ [63].

To recognize the temperature of brightness (Tb) in the Kelvin scale, a specified procedure was developed (Eq. (4)). The formula was used to calculate the Tb in Kelvin for the alteration of the thermal values of dissimilar Landsat satellite datasets [64].

(4) $$Tb=\frac{K2}{\left(\frac{K1}{L\lambda }+1\right)}$$

where K1 is a calibration constant 1 (607.76), K2 is calibration constant 2 (1260.56), and Tb is the surface temperature, which was calculated using satellite data (in Kelvin).

The transformation of Kelvin to Celsius (°C) is an additional and significant part of LST generation. To obtain the LST in Kelvin, the formula in Eq. (5) is used.

(5) LST=Tb−273.15

#### 3.4.2 Landsat 8 OLI/TIRS

The preparation of LST maps from the Landsat 8 TIRS sensor involves the conversion of the DNs of ground objects to spectral radiance using Eq. (6) [[65], [66], [67]].

(6) $$L_{\lambda }=\frac{L_{\max}-L_{\min}}{{Qcal}_{\max}-{Qcal}_{\min}}*\left(DN-{Qcal}_{\min}\right)+L_{\min}$$

where $L_{\lambda }$ is represent the top-of-atmosphere (TOA) spectral radiance in W/(m^2^.sr.μm), Qcal denotes the quantized calibrated pixel value in DN, $L_{\min}$ and $L_{\max}$ indicate the minimum and maximum spectral radiances in W/(m^2^.sr.μm), which are scaled to ${Qcal}_{\min}$ and ${Qcal}_{\max}$, respectively. ${Qcal}_{\min}$ and ${Qcal}_{\max}$ denote that the minimum and maximum quantized calibrated pixel value (corresponding to $L_{\max}$) in DN = 255.

The radiance value map projected using Eq. (9) was used to compute the brightness temperature (BT) map, allowing for the perception of black body radiation, as shown in Eq. (7) [66,68].

(7) $$T_{B}=\frac{K_{2}}{\ln\;\;\ln\left(\frac{K_{1}}{L_{\lambda }}+1\right)}-273.15$$

where $T_{B}$ indicates the operative satellite BT in degrees Celsius, $L_{\lambda }$ denotes the spectral radiance, and $K_{1}$ and $K_{2}$ represent pre-calibration constants attained from the satellite metadata files.

The subsequent stage was to precisely determine the satellite BT using surface emissivity alteration before procuring the LST map of the city [69,70]. In this study, the method of Sobrino et al. (2004) was used to calculate the results, which included the estimation of the standard deviation (m), combined soil and vegetation emissivity (n), and proportion of vegetation ($P_{V}$), as calculated from Eqs. (8), (9), (10). These three structures were used to obtain the surface emissivity from Eq. (11).

(8) $$m=\left(\varepsilon_{v}{-\varepsilon }_{S}\right)-\left({1-\varepsilon }_{S}\right)F\varepsilon_{v}$$

(9) $$n=\varepsilon_{S}+\left({1-\varepsilon }_{S}\right)F\varepsilon_{v}$$

(10) $$P_{V}={\left(\frac{NDVI-{NDVI}_{\min}}{{NDVI}_{\max}-{NDVI}_{\min}}\right)}^{2}$$

(11) $$\varepsilon =mP_{V}+n$$

where $\varepsilon_{v}$ and $\varepsilon_{S}$ indicate the vegetation and soil emissivity, respectively, and F is the figure factor (=0.55) of the observed miscellaneous geometric distribution [71]. The values of m and n are measured as 0.004 and 0.986, respectively [71]. The NDVI map was obtained using Eq. (13), as described in Section 3.5.

The final LST map was obtained using Eq. (12) by considering the satellite BT ($T_{B}$) and surface emissivity (ε) [70,72,73].

(12) $$LST\;\left({}^{\circ}C\right)=\frac{T_{B}}{1+\left(\lambda *\frac{T_{B}}{\rho }\right)\ln\;\varepsilon }$$

where λ indicates the wavelength of emitted radiance (λ=10.8μm), $\rho =h*\frac{c}{\sigma }$ (1.438 × 10^−2^ m K), c denotes the velocity of light (2.998 × 108 m/s), σ indicates the Stefan Boltzmann constant (1.38 × 10^−23^ J/K), h denotes the Planck's constant (6.625 × 10^−34^ J s), and ε indicates the surface emissivity.

### 3.5 Geo-spatial index

Geospatial indicators have been widely applied for monitoring and assessing environmental fluctuations, ecological alteration, urban expansion, moisture content availability, and soil conditions. Vegetation is essential for managing oxygen demand, precipitation, groundwater recharge, infiltration rates, and thermal comfort. However, urbanization influences NDBI values [55,74,75]. Widespread construction, infrastructural development, and railway and road development increases surface runoff and decreases the infiltration rate; therefore, the NDMI values change over time. These indicators have previously been applied to different optical imaging techniques. These four types of indices were applied for environmental assessments of the two cities investigated in this study, from 1991 to 2021, with ten years of interval. The analyses included vegetation, urban expansion, moisture content in the soil, and SAVI to determine fluctuation in addition to land alteration measurement.

(13) $$NDVI=\frac{\left(\rho_{NIR}-\rho_{red}\right)}{\left(\rho_{NIR}+\rho_{red}\right)}$$

(14) $$NDBI=\frac{\left(\rho_{SWIR}-\rho_{NIR}\right)}{\left(\rho_{SWIR}+\rho_{NIR}\right)}$$

(15) $$SAVI=\frac{\left(1+L\right)\times \left(\rho_{NIR}-\rho_{red}\right)}{\left(\rho_{NIR}+\rho_{red}+L\right)}$$

(16) $$NDMI=\frac{\left(\rho_{NIR}-\rho_{SWIR1}\right)}{\left(\rho_{NIR}+\rho_{SWIR1}\right)}$$

Geospatial indicators such as NDVI, NDBI, NDMI, and SAVI are widely applied to generate environmental assessment parameters, where the first three parameters range from −1 to +1. Positive values denote high degrees of vegetation, settlement, and available moisture content, whereas a negative value indicates other features or low content at this location.

### 3.6 Urban heat island investigation approaches

The UHI is one of the principal difficulties accompanying the industrialization and urbanization anthropological activities. The UHI, which is modeled through thermal pressure, increases the thermal variation and aggravates the effects on human health. Consequently, the UHI has been a dominant leitmotif among researchers and climatologists and has been recognized in numerous cities and metropolitan zones worldwide [53,73,76]. UHIs are categorized based on the augmented temperature, and they can hypothetically intensify the duration and magnitude of heat waves surrounding cities. Scientists have shown that the impact of heat waves on individuals fluctuates in districts with cities. The UHI effect is an alteration in temperature in cities or megacities and their neighboring rural or fringe regions. Urbanization and meteorological influences cause the UHI effect through the intensification of heat-related temperatures owing to the electricity mandate in urban areas [77]. The UHI concentration is defined as the difference between the normal temperature in the UHI area and that in the rural/fringe expansion. The 4–5 TM and 8 OLI/TIRS datasets were applied to identify UHIs, particularly in the satellite thermal infrared bands. A negative UHI indicates that the temperature in the urban area is lower than that in the rural/fringe area, and thus it is designated as an urban cool island. The UTFVI is the most frequently used index in surface UHI examinations [53]. Numerous researchers and scientists have applied this method to analyze the urban ecology, SUHI intensity, and health effects. The thermal heat difference and UTFVI evidence are valuable for producing complete information about the ecological alteration derived from climate change and anthropogenic activities. This information is useful for smart urban planning, design, and management decisions.

The LST evidence can be used for obtaining altered thermal comfort-related information and urban heat balance indicators, such as the UTFVI. It allows identifying ecological disturbances because of thermal variation and is characterized as a heat island-related examination of the urban or surrounding area [78]. Urbanization considerably affects LULC, which determines the UHI. The UHI influence is identified by associating the temperatures in the urban/rural/fringe area with those in the immediate neighborhood. The significance of UHIs has attracted growing attention in research on ecological alteration and urban environment. Numerous approaches have been used in various countries worldwide to control the magnitude and intensity of UHIs [38]. The development of RS methods has been established as a useful technique in UHI investigations. The UHI can be examined and explored by applying multitemporal thermal RS datasets.

(17) $$UTFVI=\left(\frac{T_{kelvin}-T_{mean}}{T_{mean}}\right)$$

(18) $$UHI=\left(\frac{T_{kelvin}-T_{mean}}{T_{SD}}\right)$$

where $T_{kelvin}$ represents the thermal diagrams in degrees Kelvin, $T_{mean}$ signifies the LST mean value map, and $T_{SD}$ denotes the LST standard deviation value map. These maps are valuable for approximating of the UHI dissimilarity properties and ecological disturbances. The UTFVI standards are separated into six parts, as listed in Table 3.

**Table 3:** Scale and ecological evaluation index values of urban thermal field variation index (UTFVI).

Urban thermal field variation index	Urban thermal island phenomenon	Ecological evaluation index
<0	None	Excellent
0–0.005	Weak	Good
0.005–0.010	Middle	Normal
0.010–0.015	Strong	Bad
0.015–0.020	Stronger	Worse
>0.020	Strongest	Worst

### 3.7 Applications of road network analyses

#### 3.7.1 Road density analysis

Road network density is important for examining the compact nature of road areas or transportation in specific expanses or cities. This research examined both point and line density, which are practical towards computing the road network density and traffic accident point density [79]. The point density standard determines, and the standard line density computes, the line segment length in a particular unit of a zone and the entire statistics values in the components of a part. For example, to capture the point density of accidents, some cities are divided into numerous minor right-angled cells through the adjacent measurement of *d* (conforming a pixel-oriented component in a GIS map). The density of provincial accidents characterized by the cell *k* is *Drk, Dak* denotes the density of the road network, and the neighborhood radius is set as *ρ*, *Nk* (*ρ*) represents the number of accidents happening in cell *k* defined by a center point and a radius *ρ*, and *Lk* (*ρ*) represents the measurement of roads in an equivalent neighborhood. *Drk* and *Dak* are defined by Eq. (19).

(19) $$\left[D_{1}^{a}D_{2}^{a}\ldots ..D_{k}^{a}\right]=\frac{1}{{\pi \rho }^{2}}\left[N_{1}\left(\rho \right)\;N_{2}\left(\rho \right)\ldots ..N_{k}\left(\rho \right)\right]\;\left[D_{1}^{r}D_{2}^{r}\ldots ..D_{k}^{r}\right]=\frac{1}{{\pi \rho }^{2}}\left[L_{1}\left(\rho \right)\;L_{2}\left(\rho \right)\ldots ..L_{k}\left(\rho \right)\right]$$

The density calculation was performed for every cell using Eq. (19), and finally the researchers obtained an accident-prone dissemination diagram of the density. An investigation of the concentration of road linkages was an individual requirement to substitute the investigation of accident-prone points on the road segment. Most of the aforementioned traffic accident investigations have concentrated on accident point frequency [[80], [81], [82], [83]]. The occupancy of the *l*th severity accident that occurs in the neighborhood of cell *k* is *xl*, *l* = 1, 2, …, *Nk* (*ρ*), where the accident severity density (SD) of cell *k* is denoted as *Ds k,* as expressed by Eq. (20).

(20) $$\left[D_{1}^{a}D_{2}^{a}\ldots ..D_{k}^{a}\right]=\frac{1}{{\pi \rho }^{2}}\left[\sum_{l=1}^{N_{1\;\left(\rho \right)}}x_{l}\sum_{l=1}^{N_{2\;\left(\rho \right)}}x_{l}\ldots ..\sum_{l=1}^{N_{k\;\left(\rho \right)}}x_{l}\right]$$

#### 3.7.2 Kernel density estimation (KDE)

KDE has mostly been applied to the investigation of density-related information. In road and accident-prone area analyses, the KDE is widely used in spatial modeling. The KDE approach was applied in a bandwidth study for creating a smooth surface for any category of density estimation. Subsequently, the importance was maximum at the point-event center and it reduced gradually to zero in the range of the investigation circle. In conclusion, a smooth superficial concentration was obtained through the addition of separate kernels in the investigated section [41]. The following equation is used for the KDE.

(21) $$f\left(s\right)=\frac{1}{{nh}^{2}}\sum_{i=1}^{n}K\left(\frac{d_{i}}{h}\right)$$

where f(s) is the density estimation of a particular section of s; n is the number of observations, *h* is the investigation bandwidth, *K* is the kernel function, and $d_{i}$ is defined as the remoteness of the actual position *s* in the ith observation.

#### 3.7.3 Hotspot measurement

Hotspot analysis is mostly used in investigations of highly affected areas, where actual datasets are more important; otherwise, road accident or safety identification do not examine hotspot areas [84]. Hotspots were established at equal intervals, and very low, low, medium, high, and very high accident-prone zones were determined [85,[86], [87]]. The polygon, line, and point datasets were applied for hotspot examination in different software packages, and the hotspot area was also manually identified.

(22) $$G_{i}^{*}\left(d\right)=\frac{\sum_{j=1}^{n}W_{ij}\left(d\right)x_{j}-w_{i}^{*}\bar{x}}{s{\left[\frac{W_{i}^{*}(n-W_{i}^{*}}{n-1}\right]}^{\frac{1}{2}}}$$

where x is the probability of the spatial data, $W_{ij}$ indicates the spatial weight of the selected datasets, which varies between j and i; $W_{ij}$ is the weighted sum $of\;W_{i}$; s represents the standard deviation of the *x* values, and $\bar{x}$ indicates the mean values of the selected datasets.

#### 3.7.4 Shortage distance analysis

Point-to-point analysis is the most important method used in road network analyses and other road-related activities. Some important methods, such as finding the nearest, shorter distance, and fastest route identification, are based on point-to-point analysis. Shortage distance is another method wherein shortage paths or roads from one place to another are estimated using network analysis tools. Shortage distance is widely used for fastest distance analysis and in different methods to reach a destination. The shortage-distance method uses artificial intelligence (AL) to investigate node-based road identification.

## 4 Results and discussion

### 4.1 Decadal LULC investigation and analysis

Land change analysis is a critical issue due to unplanned urbanization, industrial growth, transportation development with demand, and anthropogenic events. Currently, the world's cities are planning new strategies for achieving healthy livelihoods as well as controlling the heat island effects on deforestation. Research on land transformation is important for the identification of the Earth's surface change and for analysis of the climatic and ecological alteration, extreme weather, and environmental degradation. Because of overwhelming population pressure, unplanned urban expansion and transportation improvements are impacting the natural environment. Therefore, novel strategies, smart city-related adaptation policies, and awareness planning are very important for achieving a sustainable urban development and reducing the related ecological alteration. The LULC of the two studied cities was calculated using Landsat TM and OLI/TIRS datasets with 30 m resolution. Kuala Lumpur and Madrid are in Malaysia and Spain, respectively. Data from 1991 to 2021 with 10 years of interval were applied for both cities. Kuala Lumpur is mostly affected by residential land expansion due to high population growth and migration of foreign workers. Therefore, the greater Kuala Lumpur city comprises highly built-up lands, as observed from space-based satellite datasets. This area was mostly covered by dense forest land; however, urban expansion has reduced forest land and increased residential areas throughout the greater Kuala Lumpur. The classification information indicates that the built-up land increased by approximately 685.56 km^2^ (1991), 998.05 km^2^ (2001), 1269.67 km^2^ (2011), and 1507.41 km^2^ (2021). This dataset indicates that the proportion of construction area has increased from 22.63% to 49.75% in 30 years, particularly in the western, southern, and northern parts of this city. The Kapar, Pulau Indah, Carey Island, Selayang, Wangsa Maju, and Setia Alam areas showed noticeable changes from vegetation to built-up land (Table 4). Kuala Lumpur city has a high rate of loss of dense vegetation due to urban expansion and unexpected population growth. The Kampung Padang, Sungai Tekala recreational forest and Hulu Langat forest exhibit remarkable changes as can be observed from the Earth's observational satellite datasets. From the classification outcomes, the dense vegetation areas were measured as 2070.61 km^2^ (1991), 1699.23 km^2^ (2001), 1407.81 km^2^ (2011), and 1185.34 km^2^ (2021). During 30 years, the dense vegetation area decreased from 68.34% to 39.12% of the total land. The agricultural land, measured at different times, occupied 180.14 km^2^ (1991), 208.34 km^2^ (2001), 220.38 km^2^ (2011), and 184.16 km^2^ (2021), as presented in Table 4. Especially, the eastern and north-eastern parts of Kuala Lumpur contain agricultural land. Water bodies have increased slightly in the study area, with areas of 77.26 km^2^, 84.96 km^2^, 88.67 km^2^, and 90.53 km^2^ from 1991 to 2021, with intervals of ten years (Fig. 3a). These changes indicate that the city has experienced huge land alterations with high environmental impacts. Some studies on temperature variation, heat island effects, vegetation status, and moisture content measurement have described the conditions of the area and effects of thermal variation over Kuala Lumpur. Therefore, geospatial indices, LST, and other aspects should be investigated. Consequently, knowledge on the different land types, in addition to their spatial distribution in maps, is required for their management, and geospatial planning is necessary for the optimum management of the land resources. Information about the spatial LULC pattern in the urban areas is needed to understand its environmental impact.

**Table 4:** Area calculation of the two different city.

Kuala Lumpur								
Class Name	Area (km^2^)				Area (%)			
	1991	2001	2011	2021	1991	2001	2011	2021
Water Body	77.26	84.96	88.67	90.53	2.55	2.80	2.93	2.99
Densely Vegetation	2070.61	1699.23	1407.81	1185.34	68.34	56.08	46.46	39.12
Grass/Open Forest	16.43	39.42	43.47	62.56	0.54	1.30	1.43	2.06
Built-up Land	685.56	998.05	1269.67	1507.41	22.63	32.94	41.90	49.75
Agricultural Land	180.14	208.34	220.38	184.16	5.95	6.88	7.27	6.08
**Madrid**								
Class Name	Area (km^2^)				Area (%)			
	1991	2001	2011	2021	1991	2001	2011	2021
Water Body	5.56	6.47	6.21	6.65	0.32	0.37	0.35	0.38
Built-up Land	258.66	378.13	583.13	662.75	14.70	21.50	33.15	37.68
Densely Vegetation	974.77	941.55	905.33	865.82	55.42	53.53	51.47	49.22
Grass/Open Forest	9.55	10.31	7.35	8.41	0.54	0.59	0.42	0.48
Agricultural Land	510.46	422.54	256.98	215.37	29.02	24.02	14.61	12.24

**Fig. 3a:**
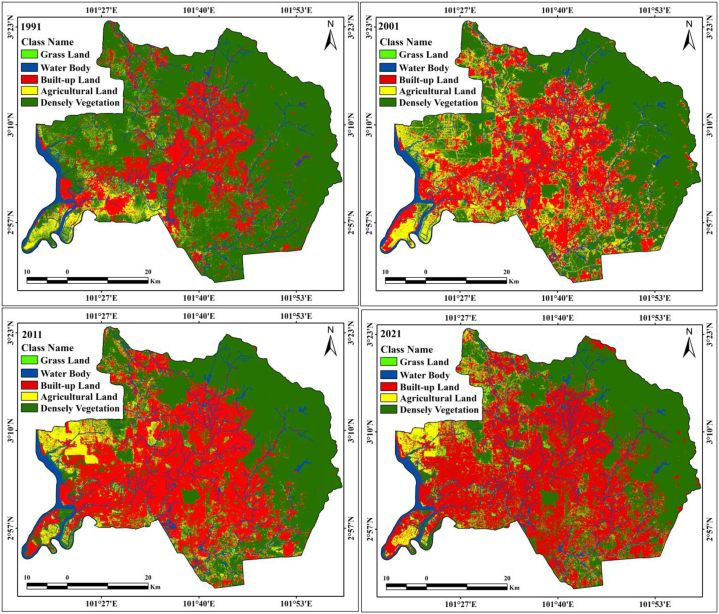
LULC classification maps of Kuala Lumpur from 1991 to 2021.

Not only population growth and climate change cause land alteration. Transportation development is also important for land alteration and thermal variation. Madrid was also affected by urban expansion and high levels of road construction. Earth observational datasets show that Madrid city has experienced a huge built-up expansion, with areas of 258.66 km^2^ (1991), 378.13 km^2^ (2001), 583.13 km^2^ (2011), and 662.75 km^2^ (2021). In 30 years, the proportion of built-up land increased by 14.70%–37.68% in the south, west, north-west, and south-west directions of the city (Table 4). Villaverde, Carabanchel, Pinar Del Rey, Hortaleza, and Vicalaro experienced noticeable urban expansion (Fig. 3b). Madrid city has huge expanses of agricultural lands in the south, west, south-west, and some portions of the eastern parts. However, urban expansion and high-road construction are triggering land alteration from agricultural lands to built-up lands in most of Madrid. In particular, croplands are being converted into uncultivated zones of built-up areas or open grasslands. In the same process, vegetation is transformed into agricultural land and built-up areas. Population growth, increasing number of urban households, transportation networks, demand for urban life activities, and gatherings have intensified the need to eliminate trees everywhere. The north, east, and some portions of the eastern lands are covered by densely vegetated lands; however, decadal datasets indicate that urban expansion has also changed forest lands and decreased green land over the study area. Classification maps indicate that the areas with dense vegetation reduced from 974.77 km^2^ to 941.55 km^2^, 905.33 km^2^, and 865 km^2^ from 1991 to 2021 with intervals of 10 years, and the proportion of forest lands reduced from 55.42% to 49.22% in just 30 years. As agricultural land gradually decreases, food scarcity-related information may help address food demand problems in these areas. Future research will be helpful for promoting sustainable food diversity in this area. The agricultural land decreased from 510.46 km^2^ to 422.54 km^2^, 256.98 km^2^, and 215.37 km^2^ in just 30 years (Table 4). This information is useful for city planners, developers, and administrative authorities in addressing future land alteration-related issues and encouraging healthy environmental development in these two cities.

**Fig. 3b:**
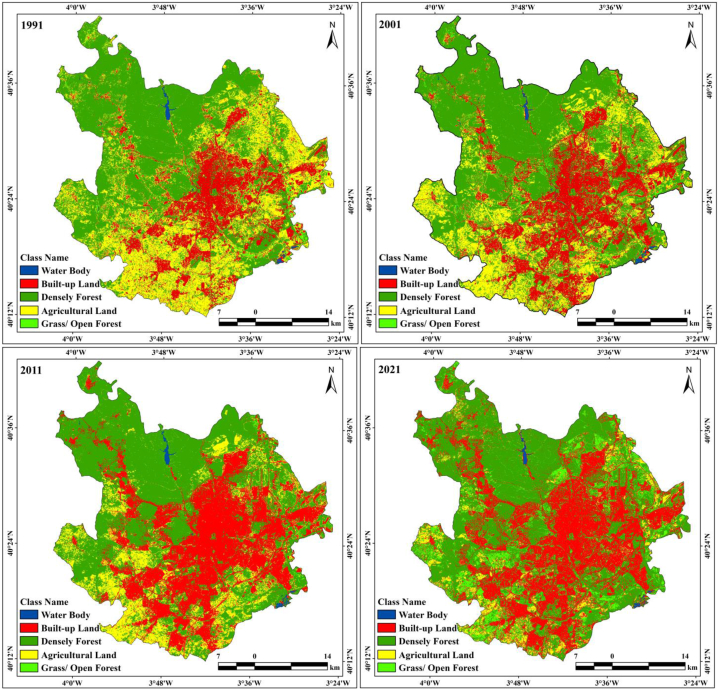
LULC classification maps of Madrid form 1991 to 2021.

### 4.2 Alteration measurement

**Fig. 4:**
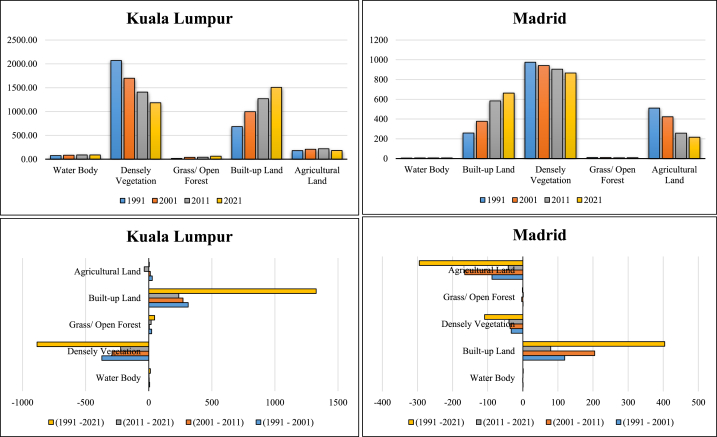
Area calculation of different LULC classes.

RS and GIS procedures are widely applied for monitoring the Earth's surface alteration and changes in different aspects, such as urban expansion, ecological alteration, thermal variation, green space dynamics, forest health, and land transformation, and also for performing observational investigations of the Earth. Research on LULC modification is very important for investigations on Earth's surface change, where loss and gain are examined and transformed locations are identified. The loss and gain of land features obtained from image classification are vital for annual changes in LULC features. Table 5 presents the land fluctuations in the two cities from 1991 to 2021. Kuala Lumpur city has had a huge increase in built-up land: 312.49 km^2^ (1991–2001), 271.62 km^2^ (2001–2011), 237.74 km^2^ (2011–2021), and 1327.27 km^2^ (1991–2021). Madrid city also had built-up expansion, as follows: 119.47 km^2^ (1991–2001), 205 km^2^ (2001–2011), 79.62 km^2^ (2011–2021), and 404.09 km^2^ (1991–2021) (Table 5). This indicates that Kuala Lumpur city experienced more built-up expansion from 1991 to 2021 (Fig. 4). Dense vegetation areas in both cities were reduced. In Madrid city, the reduction was of 33.22 km^2^ (1991–2001), 36.22 km^2^ (2001–2011), 39.51 km^2^ (2011–2021), and 108.95 km^2^ (1991–2021), whereas in Kuala Lumpur city it was of 371.38 km^2^, 291.42 km^2^, 222.47 km^2^, and 885.27 km^2^ for the same periods (Fig. 5a). Built-up land was the main reason for the overall land alteration, and Fig. 5b shows the decadal urban expansion of both cities over four different periods. Agricultural land has had a measurable change in both cities, with an increase of 4.02 km^2^ in Kuala Lumpur city while 295.09 km^2^ in Madrid city from 1991 to 2021 (Table 5). The Madrid region should be measured considering its characteristic multidimensional urban development circumstances in Europe. This multifaceted expansion reflects the fundaments of the city in relation to those of larger urban zones in the Mediterranean region. The accuracy assessment and kappa coefficients of the two selected cities, Kuala Lumpur and Madrid, are listed in Table 6, Table 7, Table 8, Table 9, Table 10, Table 11, Table 12, Table 13. The accuracy assessment and kappa coefficient were within acceptable limits, and the overall accuracy was more than 90%, which is acceptable for image classification and analysis of the LULC class for determining the decadal variation of different LULC features in the selected cities.

**Table 5:** Increase/Decrease area calculation form 1991 to 2021.

Kuala Lumpur				
Class Name	Area (km^2^)			
	(1991–2001)	(2001–2011)	(2011–2021)	(1991–2021)
Water Body	7.70	3.71	1.86	13.27
Densely Vegetation	−371.38	−291.42	−222.47	−885.27
Grass/Open Forest	22.99	4.05	19.09	46.13
Built-up Land	312.49	271.62	237.74	1327.27
Agricultural Land	28.20	12.04	−36.22	4.02
**Madrid**				
Class Name	Area (km^2^)			
	(1991–2001)	(2001–2011)	(2011–2021)	(1991–2021)
Water Body	0.91	−0.26	0.44	1.09
Built-up Land	119.47	205	79.62	404.09
Densely Vegetation	−33.22	−36.22	−39.51	−108.95
Grass/Open Forest	0.76	−2.96	1.06	−1.14
Agricultural Land	−87.92	−165.56	−41.61	−295.09

**Fig. 5a:**
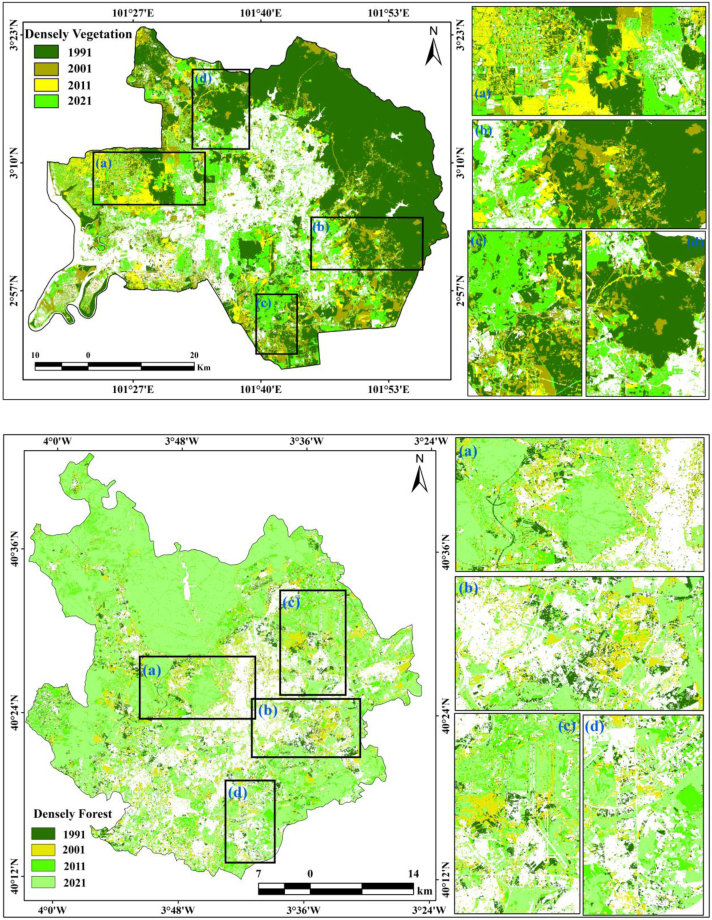
Vegetation sceneries from 1991 to 2021 for Kuala Lumpur and Madrid.

**Fig. 5b:**
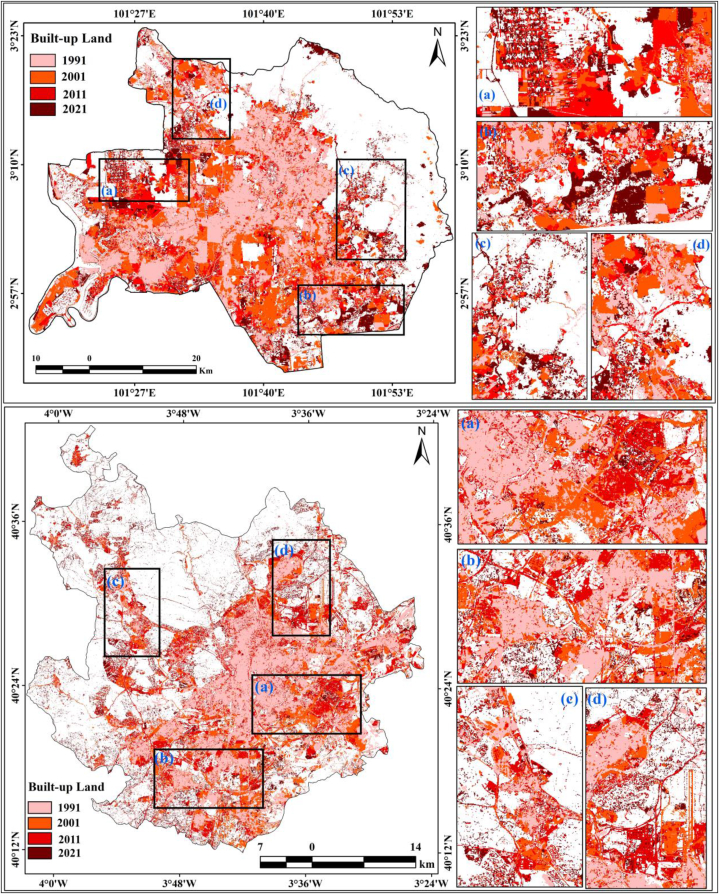
Built-up scenarios form 1991 to 2021 for Kuala Lumpur and Madrid.

**Table 6:** Accuracy assessment of Kuala Lumpur LULC for the year of 1991.

Class Name	Ground Truth/Reference					Row Total	Commission Error	User Accuracy
	Water body	Densely Vegetation	Grass/Open Forest	Built-up Land	Agricultural Land			
Water body	199	3	7	4	2	215	7.44%	92.56%
Densely Vegetation	2	331	5	7	4	349	5.16%	94.84%
Grass/Open Forest	3	4	298	2	0	317	2.84%	94.01%
Built-up Land	5	5	11	199	3	223	10.76%	89.24%
Agricultural Land	0	1	3	2	75	81	7.41%	92.59%
Column Total	209	344	324	214	84	1185		
Omission Error	4.78%	3.78%	8.02%	7.01%	10.71%			
Produce Accuracy	95.22%	96.22%	91.98%	92.99%	89.29%			
**Overall Accuracy 93% Kappa Coefficient 0.91**

**Table 7:** Accuracy assessment of Kuala Lumpur LULC for the year of 2001.

Class Name	Ground Truth/Reference					Row Total	Commission Error	User Accuracy
	Water body	Densely Vegetation	Grass/Open Forest	Built-up Land	Agricultural Land			
Water body	235	0	3	2	1	241	2.49%	97.51%
Densely Vegetation	0	359	0	10	5	374	4.01%	95.99%
Grass/Open Forest	1	1	312	7	3	324	3.70%	96.30%
Built-up Land	1	0	9	239	2	251	4.78%	95.22%
Agricultural Land	0	7	4	0	68	79	13.92%	86.08%
Column Total	237	367	328	258	79	1269		
Omission Error	0.84%	2.18%	4.88%	7.36%	13.92%			
Produce Accuracy	99.16%	97.82%	95.12%	92.64%	86.08%			
**Overall Accuracy 95.59% Kappa Coefficient 0.94**

**Table 8:** Accuracy assessment of Kuala Lumpur LULC for the year of 2011.

Class Name	Ground Truth/Reference					Row Total	Commission Error	User Accuracy
	Water body	Densely Vegetation	Grass/Open Forest	Built-up Land	Agricultural Land			
Water body	208	5	7	5	3	228	8.77%	91.23%
Densely Vegetation	2	329	5	4	2	342	3.80%	96.20%
Grass/Open Forest	7	5	310	5	2	329	5.78%	94.22%
Built-up Land	5	5	11	201	3	225	10.67%	89.33%
Agricultural Land	1	2	4	2	76	85	10.59%	89.41%
Column Total	223	346	337	217	86	1209		
Omission Error	6.73%	4.91%	8.01%	7.37%	11.63%			
Produce Accuracy	93.27%	95.09%	91.99%	92.63%	88.37%			
**Overall Accuracy 92.97% Kappa Coefficient 0.91**

**Table 9:** Accuracy assessment of Kuala Lumpur LULC for the year of 2021.

Class Name	Ground Truth/Reference					Row Total	Commission Error	User Accuracy
	Water body	Densely Vegetation	Grass/Open Forest	Built-up Land	Agricultural Land			
Water body	257	0	14	11	1	283	9.19%	90.81%
Densely Vegetation	0	435	0	12	5	452	3.76%	96.24%
Grass/Open Forest	1	2	238	7	3	251	5.18%	94.82%
Built-up Land	0	0	9	134	2	145	7.59%	92.41%
Agricultural Land	0	14	5	0	68	87	21.84%	78.16%
Column Total	258	451	266	164	79	1218		
Omission Error	0.39%	3.55%	10.53%	18.29%	13.92%			
Produce Accuracy	99.61%	96.45%	89.47%	81.71%	86.08%			
**Overall Accuracy 92.94% Kappa Coefficient 0.91**

**Table 10:** Accuracy assessment of Madrid LULC for the year of 1991.

Class Name	Ground Truth/Reference					Row Total	Commission Error	User Accuracy
	Water Body	Built-up Land	Densely Vegetation	Grass/Open Forest	Agricultural Land			
Water Body	33	1	0	2	2	38	13.16%	86.84%
Built-up Land	0	42	1	2	4	49	14.29%	85.71%
Densely Vegetation	0	2	88	3	1	94	6.38%	93.62%
Grass/Open Forest	1	0	1	59	2	63	6.35%	93.65%
Agricultural Land	1	1	1	0	38	41	7.32%	92.68%
Column Total	35	46	91	66	47	285		
Omission Error	5.71%	8.70%	3.30%	10.61%	19.15%			
Produce Accuracy	94.29%	91.30%	96.70%	89.39%	80.85%			
**Overall Accuracy 94.38% Kappa Coefficient 0.93**

**Table 11:** Accuracy assessment of Madrid LULC for the year of 2001.

Class Name	Ground Truth/Reference					Row Total	Commission Error	User Accuracy
	Water Body	Built-up Land	Densely Vegetation	Grass/Open Forest	Agricultural Land			
Water Body	38	0	1	3	1	43	11.63%	88.37%
Built-up Land	1	46	0	3	2	52	11.54%	88.46%
Densely Vegetation	0	1	83	2	3	89	6.74%	93.26%
Grass/Open Forest	0	2	1	64	3	70	8.57%	91.43%
Agricultural Land	2	1	2	3	59	67	11.94%	88.06%
Column Total	41	50	87	75	68	321		
Omission Error	7.32%	8.00%	4.60%	14.67%	13.24%			
Produce Accuracy	92.68%	92.00%	95.40%	85.33%	86.76%			
**Overall Accuracy 93.14% Kappa Coefficient 0.92**

**Table 12:** Accuracy assessment of Madrid LULC for the year of 2011.

Class Name	Ground Truth/Reference					Row Total	Commission Error	User Accuracy
	Water Body	Built-up Land	Densely Vegetation	Grass/Open Forest	Agricultural Land			
Water Body	26	1	2	2	0	31	16.13%	83.87%
Built-up Land	2	42	1	2	1	48	12.50%	87.50%
Densely Vegetation	1	2	79	3	1	86	8.14%	91.86%
Grass/Open Forest	1	1	2	59	2	65	9.23%	90.77%
Agricultural Land	1	2	0	4	42	49	14.29%	85.71%
Column Total	31	48	84	70	46	279		
Omission Error	16.13%	12.50%	5.95%	15.71%	8.70%			
Produce Accuracy	83.87%	87.50%	94.05%	84.29%	91.30%			
**Overall Accuracy 90.32% Kappa Coefficient 0.88**

**Table 13:** Accuracy assessment of Madrid LULC for the year of 2021.

Class Name	Ground Truth/Reference					Row Total	Commission Error	User Accuracy
	Water Body	Built-up Land	Densely Vegetation	Grass/Open Forest	Agricultural Land			
Water Body	34	0	3	1	1	39	12.82%	87.18%
Built-up Land	1	49	2	1	3	56	12.50%	87.50%
Densely Vegetation	2	1	59	1	2	65	9.23%	90.77%
Grass/Open Forest	0	0	3	46	1	50	8.00%	92.00%
Agricultural Land	1	2	1	2	43	49	12.24%	87.76%
Column Total	38	52	68	51	50	259		
Omission Error	10.53%	5.77%	13.24%	9.80%	14.00%			
Produce Accuracy	89.47%	94.23%	86.76%	90.20%	86.00%			
**Overall Accuracy 91.89% Kappa Coefficient 0.90**								

### 4.3 Analysis of environmental issues with geospatial information

Deforestation analyses are also important for scrutinizing the anthropogenic activities and extreme weather conditions on the Earth's surface. Unanticipated urban expansion and unintentional urbanization have persuasive influences on vegetation health and deforestation, which directly influence environmental degradation [13,59]. The consequences of the rapid expansion of urban or built-up areas on natural land cover in terms of farmland and forest are noticeable in many cities worldwide. Urbanization or the rapid increase and expansion of cities or built-up areas lead to the conversion of natural land into uses associated with a growing population [[88], [89], [90]]. Consequently, the investigation of vegetation health and forest degradation is complemented by information on climate and anthropogenic events in urban environments. Vegetation areas are also important for generating sustainable livelihoods, analyzing the urban green space, and decreasing thermal differences, and satellite-based catalogs have been extensively applied for examining vegetation conditions and vegetation health. NDVI was applied for decadal vegetation monitoring of this location, where forested lands and grassland had high positive values, while built-up lands and water bodies were observed as low positive to negative values. Four years of monitoring of the NDVI showed a gradual decrease in values in Kuala Lumpur city, 0.78 (1991), 0.75 (2001), 0.68 (2011), and 0.59 (2021), owing to the high built-up expansion and deforestation with road construction (Fig. 6a). In the initial period (1991), in Kuala Lumpur city, approximately 50% was forest land, but urbanization triggered deforestation and increased high-rise buildings, construction areas, and transportation development. Kuala Lumpur city has more green space than Madrid city because there are fewer forest lands in Madrid, but vegetation is still lost due to the unexpected urban expansion throughout the city. The NDVI values in Madrid are 0.77 (1991), 0.65 (2001), 0.58 (2011), and 0.44 (2021). Most of the northern part of Madrid has experienced vegetation damage. Urban expansion has triggered deforestation in addition to high building construction and transportation development; therefore, another geospatial index, NDBI, was also calculated for the two cities. The NDBI information indicates that the two cities gradually experienced high built-up expansion from 1991. The built-up index values for Kuala Lumpur city are 0.43 (1991), 0.59 (2001), 0.68 (2011), and 0.86 (2021), whereas those for Madrid city are 0.51 (1991), 0.56 (2001), 0.61 (2011) and 0.76 (2021), as shown in Fig. 6b. This indicates that urban expansion has triggered many issues in these two cities.

**Fig. 6a:**
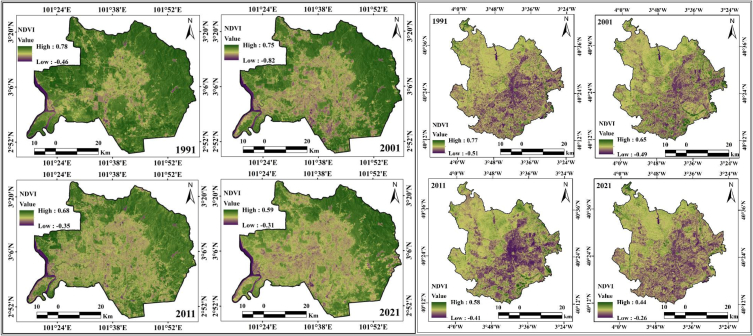
NDVI maps for two cities from 1991 to 2021.

**Fig. 6b:**
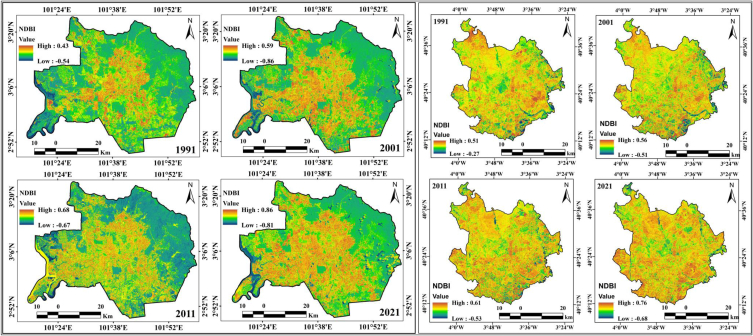
NDBI maps for two cities from 1991 to 2021.

High deforestation and urban expansion trigger low water content in the soil. Therefore, another geospatial index, NDMI, was calculated to analyze the variation in moisture content in the soil. The NDMI values of the two cities are different. In Kuala Lumpur city, the moisture index values are 0.86, 0.81, 0.66, and 0.54 from 1991 to 2021, with intervals of ten years. The moisture value decreased 0.32 in thirty years. This was caused by the loss in forest area in the Kuala Lumpur study area. Similarly, moisture values are decreasing in Madrid, where the values of the moisture index were 0.68, 0.51, 0.43, and 0.32 from 1991 to 2021 (Fig. 6c). Soil and vegetation areas are interconnected, and the vegetation depends on the soil circumstances, the accumulation of soil loss, and the vegetation conditions. The low vegetation may be caused by a low infiltration rate, high surface runoff, thermal variation, soil erosion, and soil moisture loss. The loss of soil affects the vegetation health quality, contributes to urban flooding because of the high runoff, and causes dryness of the soil. Soil adjacent vegetation (SAVI) was applied for observing the interrelation of soil and vegetation. Another important vegetation and soil condition monitoring index is SAVI, which was also calculated to compare the two selected cities. The SAVI index values of Kuala Lumpur city are 1.16, 1.13, 1.02, and 0.89 from 1991 to 2021, with intervals of ten years (Fig. 6d). Similarly, Madrid city has the same condition, and the observed SAVI values are 1.48, 1.16, 0.96, and 0.84 from 1991 to 2021. Both cities have clear fluctuations in the SAVI values: 0.27 in Kuala Lumpur and 0.64 in Madrid. This comparison indicates that these two cities, with different climatic zones, are affected by heat island effects. The datasets for the study period present different parameters for determining the impact of urban expansion on different LULC features, whereas the heat island effects trigger processes of environmental degradation in addition to ecological alteration in these cities.

**Fig. 6c:**
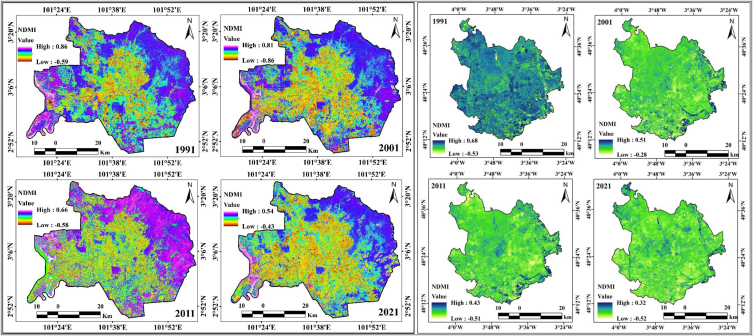
NDMI maps for two cities from 1991 to 2021.

**Fig. 6d:**
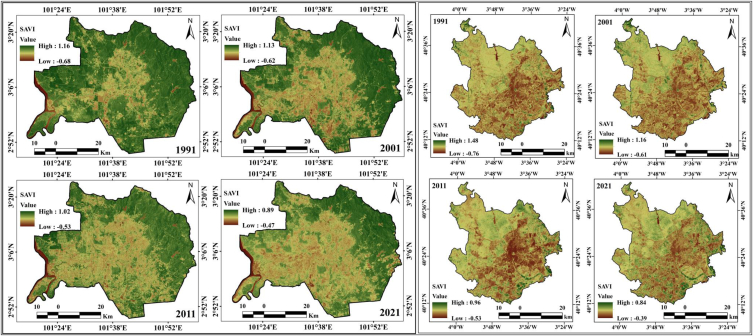
SAVI maps for two cities from 1991 to 2021.

### 4.4 Decadal thermal condition inspection

**Fig. 7:**
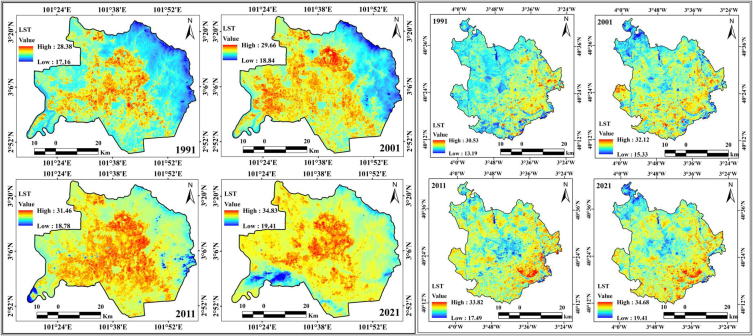
LST maps for two cities from 1991 to 2021.

The increase in global temperatures has resulted in increased rate of evapotranspiration, water shortage, fluctuations in vegetation health, and deteriorated moisture content in the soil. Temperature differences are a decisive standpoint for investigating the ecological modification, which mostly influences the external environment of the biosphere. Urban growth and anthropogenic events are principally responsible for the massive loss of vegetation and soil to roadways, construction buildings, and shadowy exteriors with urban materials (concrete, metal, and asphalt). To regulate the association between LST and numerous LULC feature categories, the mean LST was determined by overlaying LULC maps on LST images. The LSTs of these two cities were calculated using well-known formulas. The LST fluctuated as follows: 28.38 °C (1991), 29.66 °C (2001), 31.46 °C (2011), and 34.83 °C (2021). Therefore, the highest temperature of Kuala Lumpur city increased by 6.45 °C throughout the study period or 0.215 °C annually (Fig. 7). The mean temperature of Kuala Lumpur city varied as follows: 22.77 °C, 24.25 °C, 25.12 °C, and 27.12 °C, from 1991 to 2021, with intervals of ten years (Table 14). The city center locations are the most affected, with high temperature variation. Built-up locations in the center, south, north-east, and some portions of the east showed high-temperature variations with a high rate of construction and deforestation in Kuala Lumpur city. The Sentul, Taman Tun Dr Ismail, Cheras, Taman Connaught, and Subang Jaya areas showed more temperature variation due to the high vegetation damage, road construction, and infrastructural development in these locations. Space-based temperature variation analysis is essential for measurement of the ecological alteration and heat island effects throughout the study area, where LST information provides a wide angle of the measuring parts of UHI effects. Kuala Lumpur city is more urbanized, with a high rate of urban expansion in forest lands; therefore, the heat island effects gradually increase, and deforestation increases the high rate of ecological fluctuation, with variations in flora and fauna. Proper planning is required to mitigate this phenomenon. UHI effects, reduced natural vegetation coverage, and increased LST are frequently recognized as consequences of built-up expansion and growth with non-natural surfaces. To know the vegetation loss and increase in daily surface temperature, it is necessary to know and analyze the spatial trend of built-up areas. The other part of the study area is Madrid, where urban planning is implemented, but highly constructed building areas are gradually becoming harmful to the atmosphere. According to Landsat TIRS-based observations, the highest recorded LSTs were 30.53 °C, 32.12 °C, 33.82 °C, and 34.68 °C from 1991 to 2021, with intervals of ten years. The mean temperatures were 21.86 °C, 23.72 °C, 25.65 °C, and 27.04 °C. The five main LULC features, built-up land, agricultural land, dense vegetation, water bodies, and open space, influence the UHI effects, and the built-up land significantly impacts the temperature variation over the study area. The most affected areas were Alcobendas, Leganes, Chamartin, Salamanca, Atocha, and San Blas. The overall LST increase in this location was 4.15 °C with an annual interval of 0.138 °C throughout the study period. This condition indicates that the variation in LST is more harmful to the environment and local climatic conditions. It promotes the extreme climatic change and can cause problems in the future, such as increased water demand, soil moisture loss, green space transformation into building construction, and high rate of road development.

**Table 14:** Mean, maximum and minimum LST of Kuala Lumpur and Madrid.

LST (°C)												
Year	1991			2001			2011			2021		
Parameter	Mix	Min	Mean	Mix	Min	Mean	Mix	Min	Mean	Mix	Min	Mean
**Kuala Lumpur**	28.38	17.16	22.77	29.66	18.84	24.25	31.46	18.78	25.12	34.83	19.41	27.12
**Madrid**	30.53	13.19	21.86	32.12	15.33	23.72	33.82	17.49	25.65	34.68	19.41	27.04

### 4.5 Correlation analysis

Statistical analysis and multidimensional visualization of the statistical data with a scatter plot matrix are some of the most frequently used methods. This is more feasible with unambiguous variables, for which the capacity has a comparable correlation with the datasets. Correlation analysis of LST and different geospatial indices such as NDVI, NDMI, NDWI, and SAVI are essential for assessment of the impact of urbanization, deforestation, environmental impact assessment, and overall road or transportation impact on LULC features. Therefore, the selected parameters and LST information were correlated with the help of ArcGIS version 10.8, where the “create fishnet,” “extract multi values to point,” and “clipping” tools were applied for a correlation analysis. Fig. 8 shows the different correlations with LST, and Fig. 8e shows the different four-year correlations. The correlation between LST and NDBI of the two cities indicates that the correlation gradually increased in Kuala Lumpur city, with 0.38 (1991), 0.48 (2001), 0.57 (2011), and 0.61 (2021). Madrid also had an increasing LST and NDBI correlation. The correlation was 0.14 (1991), 0.25 (2001), 0.29 (2011), and 0.38 (2021). These values indicate that the urbanization process has gradually impacted the local environment. Similarly, the NDVI and LST maps indicated that the correlation between these two indicators gradually decreased. For Kuala Lumpur city, the observed correlations were 0.15 (1991), 0.27 (2001), 0.13 (2011), and 0.09 (2021). Similarly, the Madrid city correlations were 0.25 (1991), 0.14 (2001), 0.09 (2011), and 0.07 (2021). These values indicate that environmental degradation influences LST, heat variation, and vegetation damage in the two selected cities. These conditions are similarly observed for the different indices, such as NDMI and SAVI, where NDMI indicates the moisture content in the soil or Earth's surface (Fig. 8c, d). The correlation was negative for NDMI, and a similarly low negative correlation was observed for SAVI in both selected cities. These conditions indicate that urbanization, road construction, transportation development, and deforestation have triggered thermal variation and a general ecological alteration in the selected cities. Fig. 8e shows the selected overall distribution of the 60 points LST, UHI, and UTFVI values used to monitor the variation and fluctuation of the LST, UHI, and UTFVI at the same observation point. These diagrams indicate that cities have more fluctuations in the city areas and non-vegetated land due to high construction and environmental degradation.

**Fig. 8a:**
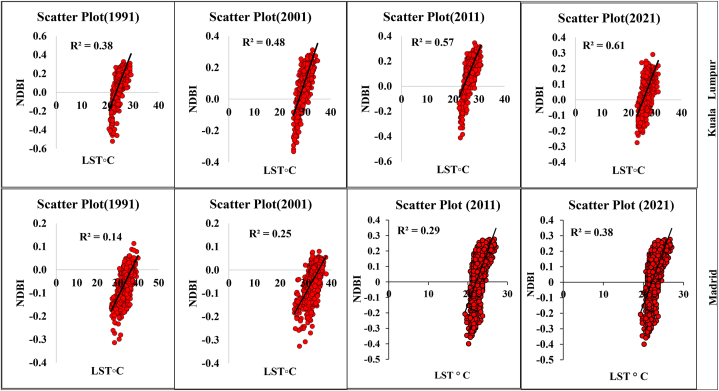
Correlation between LST and NDBI for two different cities.

**Fig. 8b:**
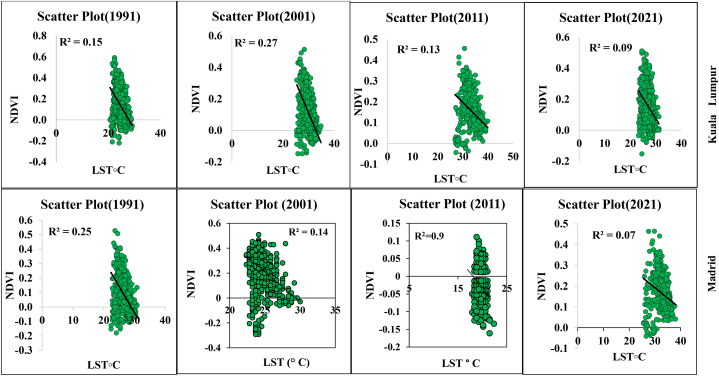
Correlation between LST and NDVI for two different cities.

**Fig. 8c:**
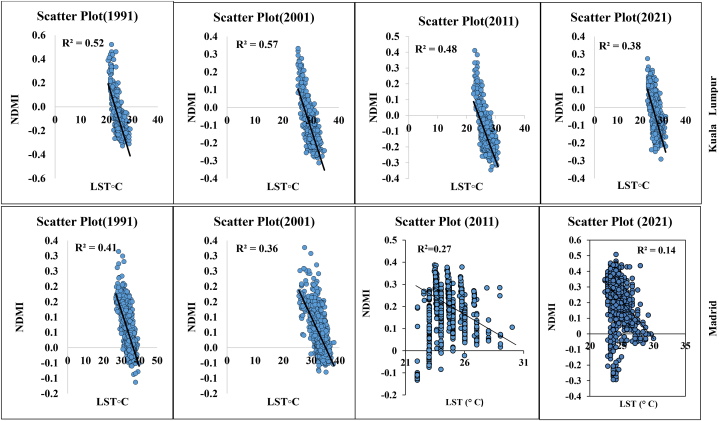
Correlation between LST and NDMI for two different cities.

**Fig. 8d:**
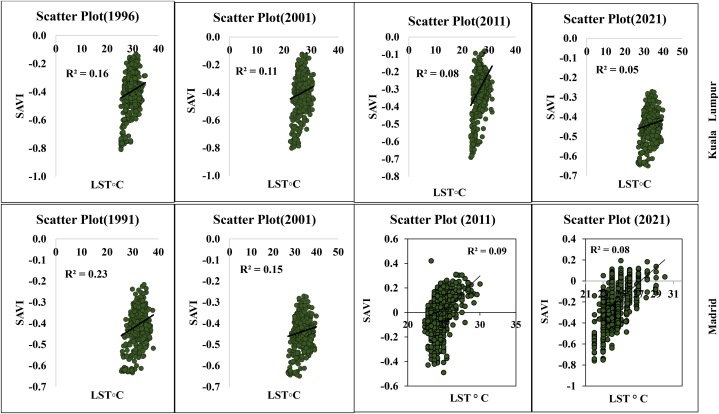
Correlation between LST and SAVI for two different cities.

**Fig. 8e:**
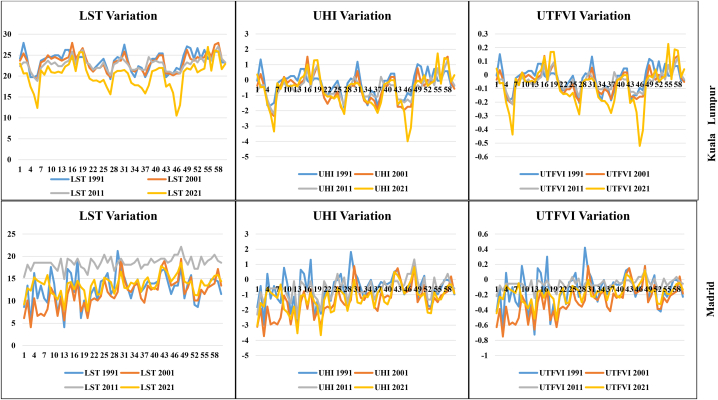
Correlation between different times LST, UHI, and UTFVI for two cities.

### 4.6 Heat island effects

The expansion of urban areas is controlled by the rapid global economic growth and intensification of the municipal population, and it causes numerous difficulties to the environmental structures. Among these difficulties, the UHI singularity, where the temperature in the urban area is significantly different from that in the rural/fringe or suburban parts, has been extensively recognized as an important urban difficulty. It causes significant issues in relation to energy, water, thermal comfort, and health conditions of the citizens. The UHI is an effective urban climate that is directly related to the two characteristics of limited environments and urban development. In the urbanization process, variations in the LULC categories are responsible for the spatial variation in the LST data. The UHIs of the two cities were observed through LST datasets using the relevant formulas, where the mean, maximum, and minimum LST information was applied. The highest values for the UHI of Kuala Lumpur city were 3.63, 4.11, 4.73, and 5.39 from 1991 to 2021, with a total increase of 1.76 during the study period and 0.059 of annual increase (Fig. 9a). Global urbanization and climate change generate impacts on megacities, towns, and city areas and are growing phenomena that trigger environmental variation and health issues. These conditions require extensive assessment and management; otherwise, climatic conditions will affect the Earth's overall surface and increase the risk of thermal variation. In addition, unplanned cities are the most affected by heat island fluctuations. This occurs because many countries have experienced unexpected urbanization, which has triggered the impact of heat islands worldwide. The UHI effects are gradually increasing throughout Kuala Lumpur city. City centers are more harmful to healthy livelihoods, and climate change is triggering other issues such as high temperatures, health issues, and low precipitation. Therefore, the UHI affects Kuala Lumpur in general. Researchers investigating the city of Madrid also observed a high rate of UHI effects, with high UHI values of 4.72, 5.61, 6.61, and 7.59 from 1991 to 2021, that is, an increase in UHI value of 2.87 over 30 years, with a 0.095 annual increase. The southern, eastern, north-western, and some portions of the middle parts of the city have significant UHI effects, as observed in the space-based Earth observational satellite datasets. The high rate of road construction and other built-up urban amenities is gradually damaging the healthy environment in both cities, and proper planning and management are important for measuring the impact of heat island effects. The outcomes of this study can help prepare planning policies that reduce the environmental variations in these study areas.

**Fig. 9a:**
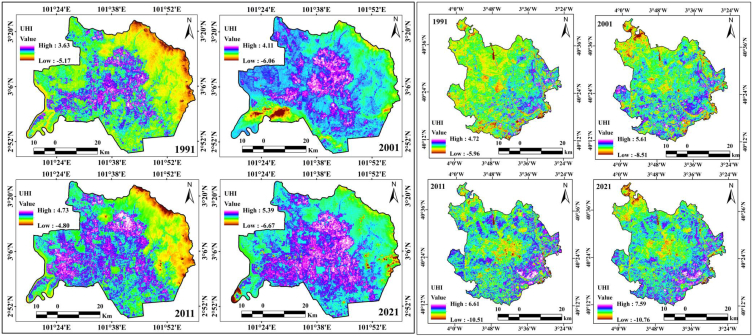
UHI maps for two cities from 1991 to 2021.

Ecological alteration was also measured in this study, where the UTFVI was calculated based on the LST in the two selected study areas, and it was observed that the UTFVI values gradually increased in both cities. The UTFVI values of Kuala Lumpur city from 1991 to 2021 were 0.39, 0.42, 0.47, and 0.52, with an increase of 0.13 in 30 years and an annual increment of 0.004 (Fig. 9b). This indicates that the UTFVI values gradually increased in Kuala Lumpur. The central parts of the city are the most affected by LST variations, in addition to ecological alteration. The forested lands of Kuala Lumpur city, located in the eastern, south-eastern, and north-eastern parts, have low UTFVI values, but the overwhelming population pressure and road network development are triggering UTFVI effects over them. Many forest land areas were converted into residential land and roads. Roads such as B116, B32, B19, B62, and B27 triggered deforestation in the study area. Madrid had the same experience with UTFVI values, which were 0.21, 0.25, 0.34, and 0.39 from 1991 to 2021, for an increment of 0.18 over 30 years, with an annual increase of 0.006. The Mingorrubio, Parque, Las Cuestas, and Castillo de Vinuelas forest areas have also been affected by the high rates of deforestation in Madrid city. These conditions indicate that these two city areas were significantly affected by heat island intensification and ecological alteration. The central parts of Kuala Lumpur city and southern parts of Madrid city require a high rate of adaptation planning and management to reduce heat island effects over the city area.

**Fig. 9b:**
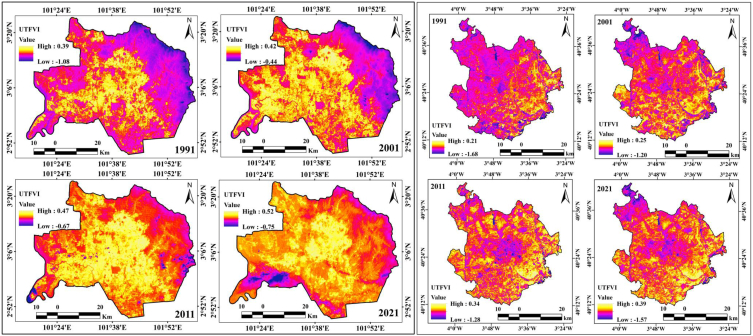
UTFVI maps for two cities from 1991 to 2021.

### 4.7 Road network assessment

**Fig. 10:**
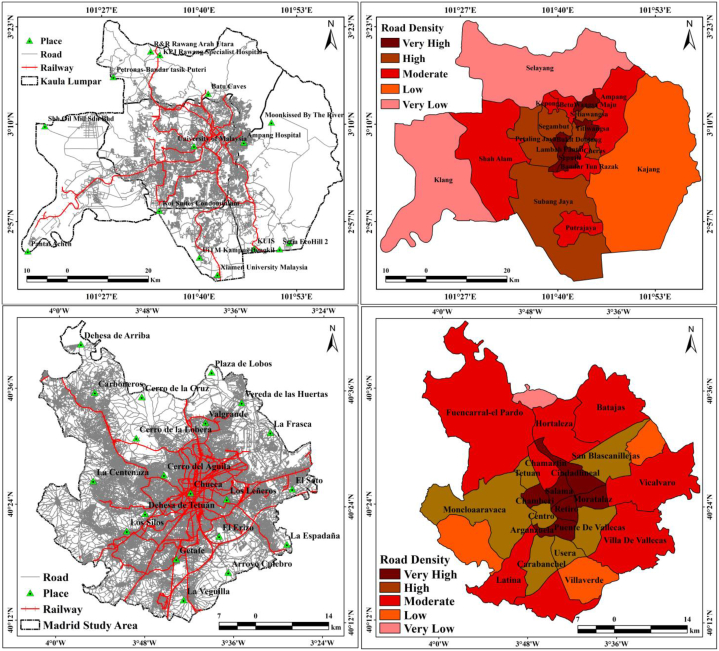
Transportation (Road and Railway) maps of two cities, and block-wise Road density.

Road network assessments focus on the contributions, characteristics, and outcomes of road networks, and are supported on the data continuously recorded on network datasets. Road network assessment based on GISs relies on the theoretical mathematical principles of graph topology. This study utilized the road route layer, and the investigation was concentrated on the issues of the road network detected in real-time and according to the road hierarchy (major roads, streets, minor roads, and railway lanes). GIS networks are composed of the lines (recognized as edges) and connections (recognized as junctions) that characterize the routes along which goods and people travel. Road network assessment assists in modeling and the preparation and organization of low-to-moderate to heavy-traffic routes. A common road network analysis category is the identification of the shortest path between two points. The interlinked edges and junctions (or nodes) have particular attributes attached to them that assist in modeling. Junctions and edges are topologically associated with other edges that need to be attached to other edges at junctions along the flow because edges in a road network are connected through junctions. Kuala Lumpur and Madrid city have large road and railway construction projects, and Chamberi, Retiro, Moratalaz, Salama, Wangsa Maju, Titiwangsa, Bukit Bintang, and Seputeh have very high road densities (Fig. 10). This indicates that the two cities have significant road-related issues, such as traffic jams, traffic accidents, and environmental problems, which lead to high LST, heat island effects, and ecological effects over the study areas. Road densities at the block scale can be categorized as very high, high, moderate, low, and very low. Therefore, the overall city parts are divided into five categories according to the road density of streets and main roads in both Kuala Lumpur and Madrid city.

#### 4.7.1 Kernel density estimation (KDE)

**Fig. 11:**
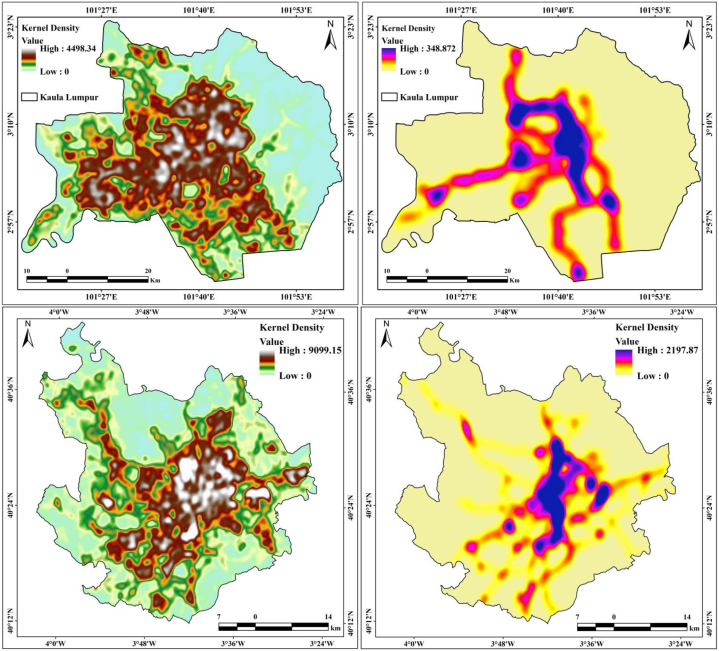
Kernel Density Estimation (KDE) of two cities (Road and Railway).

KDE computes the density in the interior of the circular distance crossed by traffic in the extent of the study area. The kernels are weighted based on their Euclidean distance from the kernel center, and the value of the resulting density is allocated to that center. KDE is vital for measuring road density and analyzing the variation in density with different parameters. The roads and railways of Kuala Lumpur and Madrid city have high traffic, and thus, KDE information is valuable for planning and management. The KDE measurements showed that the road density in Kuala Lumpur (4498.34) and Madrid (9099.15) was high in the central parts of the cities, and the railway density was 348.872 and 2197.87, respectively (Fig. 11). The two cities have different KDEs for LULC features: 62% of the road density in Kuala Lumpur is in the built-up land, 9% in dense vegetation areas, 15% in open space/grassland areas, 13% in agricultural land, and 1% in the proximity of water bodies, whereas approximately 85% of the railway density is in built-up areas. This distribution shows that the city has more transportation infrastructure in the city center area with a high rate of roads and railways. Similarly, Madrid has a high road density located in the central part of built-up areas (73%), agricultural land (11%), open spaces (9%), and vegetation areas (7%). Railway density is also high in built-up areas (80%). The KDE estimation results indicate that the density of roads and railways is a vital operational aspect for road accidents, traffic jams, and environmental impacts. Therefore, planned road and railway construction, and novel strategies for network analysis are essential for these cities.

#### 4.7.2 Hotspot measurement

**Fig. 12:**
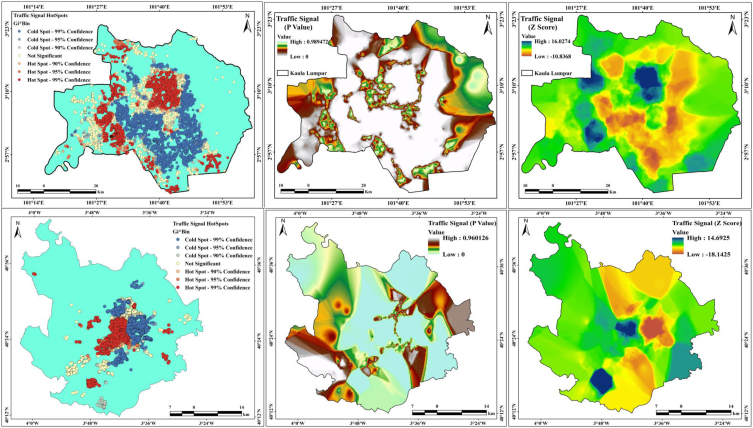
Traffic signal hotspot, *P*-values and Z-score for two cities.

**Fig. 13:**
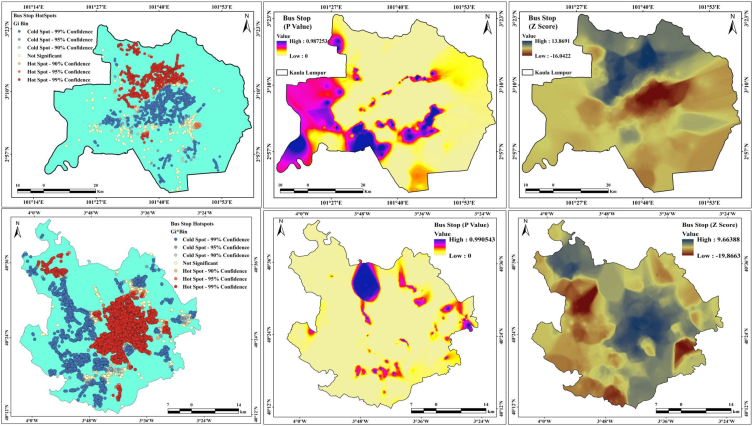
Bus stand hotspot, P-values and Z-score for two cities.

Hotspot documentation is necessary for road traffic safety programs. Locations with high concentrations of clusters of accidents are typically recognized as crash-prone points or hotspots. The number of accidents occurring at several unambiguous places or road segments throughout a reasonable historical time is a non-negative and probabilistic number. Traffic accidents are changeable at the micro level by nature. Statistical models can produce reliable approximations of predictable accident incidences as a function of descriptive variables such as site characteristics, traffic flow, and road geometry datasets at the macro level. Hotspot areas were measured because of the high level of effects on area identification, and two different hotspot maps were created: traffic signal and bus stop maps. Fig. 12 presents the traffic signal hotspots with p-values and z scores. Several hotspot areas are observed in Kuala Lumpur city, where approximately 32% of traffic signals are in hotspots areas with 99% confidence and 38% are in cold spots areas are with 99% confidence. The P values of the two cities are 0.98972 (Kuala Lumpur) and 0.960126 (Madrid), whereas the Z-score high values are 16.0274 (Kuala Lumpur) and 14.6925 (Madrid). This conditions can help reduce road accidents and traffic jams and improve the road network analysis. Madrid has more bus stands than Kuala Lumpur city, where 39% of the stands are in hotspots with 99% confidence, and 28% of the stands are in cold spots with 99% confidence (Fig. 13). The *P*-values for Kuala Lumpur and Madrid are 0.987253 (Kumpuruala L) and 0.990543 (Madrid). The Z scores of Kuala Lumpur city are 13.8691 (high) and −16.0422 (low), whereas those of Madrid city are 9.66388 (high) and −19.8663 (low). This indicates that the number of bus stands is high in Madrid and low in Kuala Lumpur city.

#### 4.7.3 Shortage distance analysis

**Fig. 14:**
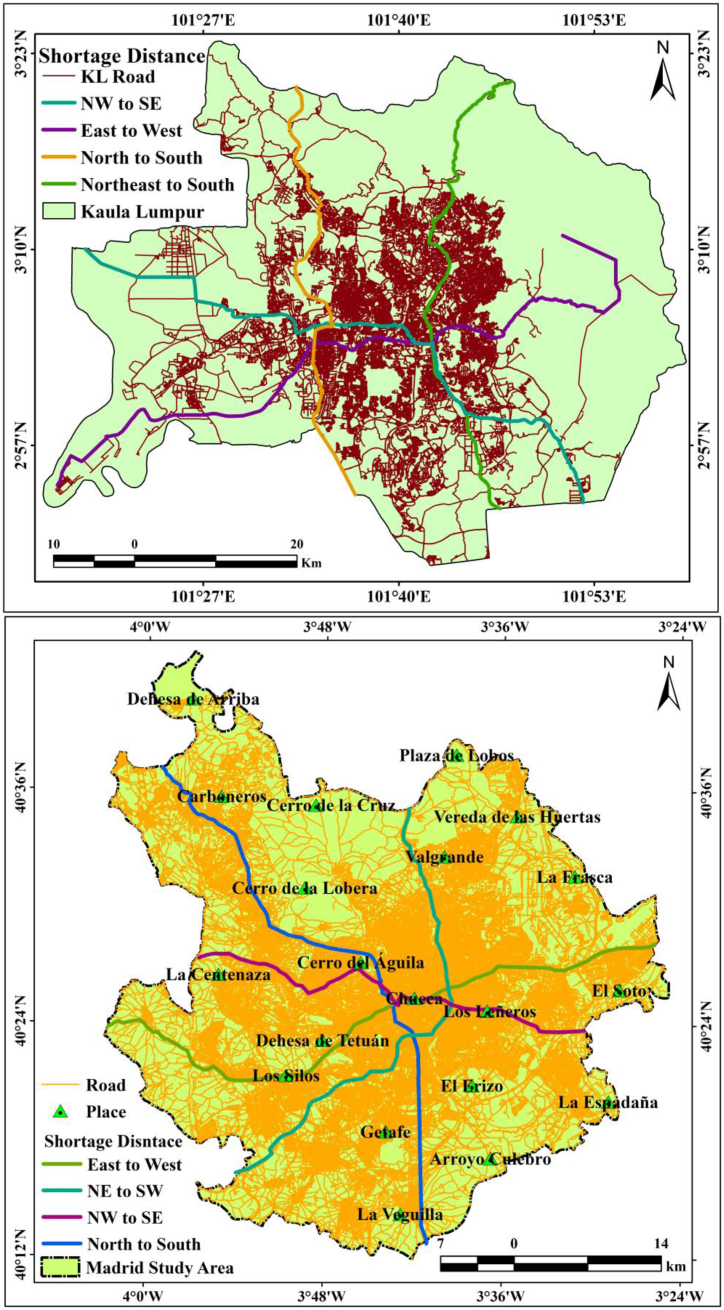
Shortage distance analysis of two cities.

Road network analysis is the most influential tool to address the actual transference difficulties in a period. This analysis is a dependable, operator-approachable, and professional way to resolve the network difficulties. It replaces conservative approaches in investigating and protecting the allocation of time and work. It serves as a pointer in transportation planning for linking origins and destinations. This analysis favors the shortest route, without considering traffic congestion, and determines the road distances. The division of dissimilar types of road traffic by substituting the shortest routes on the basis of operating characteristics and mileage of the vehicles and appropriate consideration of the transport strategy can challenge traffic congestion. Fig. 14 shows that the two studied cities have four directional shortage distances. This information can help travelers and planners to move from one part to another part of the city using GIS-based approaches.

Road/rail arrangement and environment-related assessment articles are limited. Therefore, a comprehensive assessment article can help the management team and researchers to adopt better approaches for the specific area in the world. The road network is used to move products and/or goods from facilities in one place to another, and this organization has financial requirements [91]. For example, in Africa, approximately, 75% of goods and/or passengers are transported to places in Sub-Saharan Africa [92]. The expansion of roads and the associated infrastructure is based on an approach in which 50% of the expenses are covered by the cities as road network expansion generates physical and/or social-economic development [93,94]. Ghana and Kenya have particular road network-associated approaches that are fostering growth with spatial and social heterogeneity in the peri-urban zones [95]. Analyses of variations in temperature and LULC alteration are essential for classifying the Earth's surface alteration and the associated ecological disruptions. Lands are progressively altered, and therefore, LULC analysis is required for research on alteration. In China, built-up land areas in Greater Guangzhou increased from 2001 to 2006 [96]. RS methods, the Google Earth Engine (GEE) platform, and optical image (Landsat and Sentinel-2) datasets were earlier used, but these methods have not been previously applied in the designated cities. Liu et al. (2021), used RS datasets for LULC analysis in Nanjing applying the GEE platform from 1985 to 2015 [97]. According to the results, the surface area of the urban and built-up areas of Nanjing quadrupled from 11% in 1985 to 44% in 2015. Some investigators applied Sentinel-2 datasets for LULC classification in Xiongan New Area, China, where Sentinel-2 datasets were applied for the 2016 and 2017 LULC classification, and 51.59% of the cropland was covered in this location [98]. Thermal differences were used in the examination of the Yangtze River Delta, and Landsat datasets were applied from the years 2013, 2014, and 2015 [99]. The study found that Shanghai and Hangzhou had mean LST values of 26.10 °C and 15.40 °C, respectively. Using satellite-based datasets, previous studies found that UHI and UTFVI were connected [[100], [101], [102], [103]]. The present study determined the LULC modification, ecological disturbances, thermal variation, and fluctuation of environmental parameters in the designated two cities of Malaysia and Spain using four different satellite images. The results of this analysis are valuable for scientists, disaster management teams, planners, and for awareness purposes.

The contributions of this study are the determination of decadal land alteration, identification of loss of vegetation area, analysis of heat fluctuation, and assessment of road-related issues. However, these analyses were not conducted in the same study. Both cities have experienced a huge impact on their environment owing to high population pressure, high rates of construction, vegetation damage, and gradual traffic congestion. This investigation can help reduce the effects of construction with planned urbanization, awareness to protect vegetation, use of different resources, such as electric vehicles, to reduce air pollution, and other decisions. The results of this study will also help decision-makers, administrators, and policymakers to protect these locations from extreme environmental events.

## 5 Study restrictions and recommendations

Transportation accessibility and land transformation are land-related issues. Generally, road or railway construction requires large land areas; for this reason, land alteration is a general phenomenon. In this study, road construction, density estimation, hotspot analysis, and land alteration-related issues were investigated using RS and GIS-based approaches. These methods have some limitations, such as the medium-scale resolution (30 m) of images, and therefore, appropriate land feature classification is restricted [15]. Road construction and railway lines are demarcated in a single year, and multiple years of observational data are more informative. The results of this study will be helpful for researchers, policymakers, scientists, and other stakeholders in observing and measuring climate change and anthropogenic activities, in addition to increasing the awareness of land transformation and environmental protection. Future studies are essential for a better understanding of environmental, climate-related and land-related issues, such as decadal road, highway, and bridge construction, soil moisture measurement, green space dynamics due to transportation development, and cooling space measurement over the study areas. Future studies will provide information for sustainable urban development and hustle-free transportation accessibility.

## 6 Conclusions

This paper presents a recent network analysis of two cities with land alteration impact assessments of the environment and climate. These joint techniques help prepare awareness, planning, management, and adaptation strategies to prevent future disasters. Road density and traffic accident zones are not equivalent because vehicle speed is also an important condition for road accident-related issues. Although this study has some limitations, the information it provides is useful for understanding variations in road density and traffic accidents. Road construction not only has an impact on health, but also affects the environment and climate change. Therefore, land alteration and climate change analyses were conducted in this investigation. KDE, shortage distance, hotspot measurement, Z-score, and block-wise density estimation help to understand the current situation of the spatial conditions of two different cities, Kuala Lumpur and Madrid, and increase knowledge about environmental impact and climate change issues. Kuala Lumpur, Malaysia, has low road and railway density owing to the large areas of forest and agricultural land, while Madrid, Spain has higher density of roads and railways. Both cities have a huge density in the central parts because the city centers of Madrid and Kuala Lumpur are essential. Shortage distance analysis helps avoid those city centers when moving in directions north–east, south–north, and west–east. The KDE results indicate a high density of roads and railways in Madrid (9099.15 and 2197.87, respectively). In Kuala Lumpur, the first railway between Malaya and Port Weld was officially opened on June 1, 1886, whereas in Spain, the Madrid-Aranjuez railway line was opened in 1851. After that, road and railway construction gradually increased owing to the increased demand, transportation development, and population pressure. Progressive land alterations include the construction of urban amenities and buildings, vegetation damage, and land subsidence. These phenomena are increasingly studied because of the environmental and climatic effects on these cities.

Dense vegetation areas have been reduced by 885.27 km^2^ in Kuala Lumpur and 108.95 km^2^ in Madrid. Built-up land has increased by 1327.27 km^2^ and 404.09 km^2^ in Kuala Lumpur and Madrid, respectively. In the last 30 years, the temperatures of Kuala Lumpur and Madrid have increased by 6.45 °C and 4.15 °C, respectively, due to urban expansion and road construction. Meanwhile, the agricultural land has decreased in Madrid (by 295.09 km^2^), and the cropland has been slightly reduced in Kuala Lumpur. UHI and UTFVI have had high variation in both cities: 1.76 and 0.13 in Kuala Lumpur and 2.87 and 0.18 in Madrid, respectively. This indicates that Kuala Lumpur has experienced more heat variation over the years. The results show that cities with significant heat islands are more environmentally and ecologically altered. Adaptation strategies and awareness planning are essential for reducing the effects on the environment and climate using modern transportation approaches to decrease traffic jams, traffic accidents, land alteration, and thermal variation while increasing areas with vegetation. The outcomes of this study will be helpful to local planners, policymakers, researchers, and stakeholders in adaptation planning and awareness to reduce ecological and climatic alteration. The presented methods are also useful for research in other locations, with or without further modifications.

## Author contribution statement

Khalid Hardan Mhana: Conceived and designed the experiments; Performed the experiments; Analyzed and interpreted the data; Contributed reagents, materials, analysis tools or data; Wrote the paper.

Shuhairy Bin Norhisham; Herda Yati Binti Katman; Zaher Mundher Yaseen: Conceived and designed the experiments; Analyzed and interpreted the data; Contributed reagents, materials, analysis tools or data; Wrote the paper.

## Data availability statement

Data will be made available on request.

## Additional information

Supplementary content related to this article has been published online at [URL].

## Declaration of competing interest

The authors declare that they have no known competing financial interests or personal relationships that could have appeared to influence the work reported in this paper.
